# Tumour-associated macrophages act as a slow-release reservoir of nano-therapeutic Pt(IV) pro-drug

**DOI:** 10.1038/ncomms9692

**Published:** 2015-10-27

**Authors:** Miles A. Miller, Yao-Rong Zheng, Suresh Gadde, Christina Pfirschke, Harshal Zope, Camilla Engblom, Rainer H. Kohler, Yoshiko Iwamoto, Katherine S. Yang, Bjorn Askevold, Nagesh Kolishetti, Mikael Pittet, Stephen J. Lippard, Omid C. Farokhzad, Ralph Weissleder

**Affiliations:** 1 Center for Systems Biology, Massachusetts General Hospital (MGH), Harvard Medical School, 185 Cambridge Street, Boston, Massachusetts 02114, USA; 2 Department of Chemistry, Massachusetts Institute of Technology (MIT), 77 Massachusetts Avenue, Cambridge, Massachusetts 02139, USA; 3 Laboratory of Nanomedicine and Biomaterials, Department of Anesthesiology, Brigham and Women’s Hospital (BWH), Harvard Medical School, 75 Francis Street, Boston, Massachusetts 02115, USA; 4 King Abdulaziz University, Jeddah, Saudi Arabia; 5 Department of Systems Biology, Harvard Medical School, 200 Longwood Avenue, Boston, Massachusetts 02115, USA

## Abstract

Therapeutic nanoparticles (TNPs) aim to deliver drugs more safely and effectively to cancers, yet clinical results have been unpredictable owing to limited *in vivo* understanding. Here we use single-cell imaging of intratumoral TNP pharmacokinetics and pharmacodynamics to better comprehend their heterogeneous behaviour. Model TNPs comprising a fluorescent platinum(IV) pro-drug and a clinically tested polymer platform (PLGA-*b*-PEG) promote long drug circulation and alter accumulation by directing cellular uptake toward tumour-associated macrophages (TAMs). Simultaneous imaging of TNP vehicle, its drug payload and single-cell DNA damage response reveals that TAMs serve as a local drug depot that accumulates significant vehicle from which DNA-damaging Pt payload gradually releases to neighbouring tumour cells. Correspondingly, TAM depletion reduces intratumoral TNP accumulation and efficacy. Thus, nanotherapeutics co-opt TAMs for drug delivery, which has implications for TNP design and for selecting patients into trials.

## Abstract (web summary)

Drug-loaded nanoparticles allow controlled release and enhanced delivery, yet understanding *in vivo* behavior has been difficult. Here, the authors develop a platinum prodrug coupled to a polymer platform, and use intravital imaging to show that the nanoparticle accumulates in macrophages, from the which drug redistributes to neighboring tumour cells.

## Graphical abstract

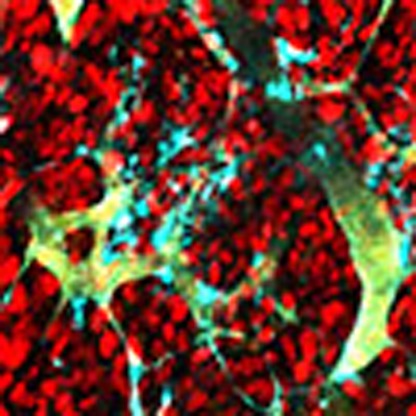

Approximately half of all cancer patients who receive chemotherapy are treated with one of three clinically approved platinum (Pt) drugs—cisplatin, carboplatin, and oxaliplatin^1^. These Pt compounds are first-line agents in ovarian, lung, head and neck and testicular cancers, among others, and development of combination therapies using Pt compounds is an active area of research^2^. Unfortunately, most cancers either exhibit intrinsic Pt resistance or ultimately develop resistance to treatment through mechanisms including reduced drug uptake, altered cellular metabolism, increased DNA repair, chemoprotective niche formation and/or activation of oncogenic and anti-apoptotic pathways^3^. Dose escalation and combination drug regimens help overcome resistance. However, Pt-related toxic side-effects including neurotoxicity and nephrotoxicity limit some of these approaches, indicating the need for new strategies.

To circumvent these problems, specialized drug-delivery mechanisms such as nanoparticles (NPs) have been introduced to enhance local drug accumulation in tumours while simultaneously mitigating systemic toxicities^4^,^5^. Non-encapsulated Pt compounds in particular are often plagued by poor pharmacokinetic (PK) properties. They generally must be intravenously administered over a prolonged period (sometimes over 24 h) because promiscuous Pt reactivity causes side effects and the covalent binding to plasma proteins such as albumin consequently neutralizes its activity. Recent work has demonstrated the possibility of using NPs to encapsulate Pt pro-drugs, thereby (i) extending the circulating half-life of the unreacted Pt compound and enabling controlled drug release^6^; (ii) facilitating precisely engineered Pt-based combination therapies through co-encapsulation with additional drugs^7^,^8^ (or small interfering RNA treatments^9^); (iii) enabling the administration of more lipophilic and newer-generation Pt compounds^10^ and (iv) modifying tissue distribution and enhancing intratumoral accumulation^6^,^9^. The last is thought to occur through enhanced permeability and retention (EPR) effects and has been termed ‘passive NP targeting’^11^. In theory, enhanced permeability of the abnormal tumour microvasculature (TMV) should allow therapeutic nanoparticles (TNPs) to enter the tumour interstitial space, whereas suppressed lymphatic filtration and increased cellular uptake should allow them to stay there^12^. Although TNPs of Pt-based anticancer agents have advanced to clinical trials^13^,^14^, results have been mixed, presumably due to heterogeneous EPR effects in different tumours combined with limited experimental data from patients on the effectiveness of this mechanism as related to enhanced drug accumulation^15^. Furthermore, the inter-related *in vivo* PK and pharmacodynamics (PD) of TNPs are more difficult to understand than those of the unencapsulated drug. TNP drug delivery is inherently a multi-step process, defined by PK of the TNP vehicle, drug release dynamics that may change depending on the *in vivo* environmental context, and PK of the Pt payload itself. Little experimental evidence exists that describes how this multi-step drug delivery sequence performs *in vivo* and within tumours, despite its critical importance to overall therapeutic outcome. This lack of understanding clearly represents a bottleneck in the design and development of more efficacious therapies.

Here we use high-resolution microscopic imaging in live tumour-bearing mice to test the hypothesis that therapeutic nano-encapsulation not only affects traditional bulk PK properties, but also significantly influences heterogeneous drug uptake and response at the single-cell level within tumours^16^,^17^. We engineer a platform that for the first time allows simultaneous imaging of both a ∼100-nm TNP vehicle as well as its Pt payload, in addition to monitoring DNA damage at the single-cell level in real time. As expected, our results show that nano-encapsulated Pt exhibits a longer circulating half-life than traditional unencapsulated Pt compounds. However, we quite unexpectedly find that TNPs accumulate at high levels within tumour-associated macrophages (TAMs), and that TAMs serve as ‘cellular drug reservoirs’. Indeed, TAMs release the Pt payload into neighbouring tumour cells over time. Depletion of macrophages significantly decreases intratumoral Pt accumulation and correspondingly increases tumour growth. Overall, this work establishes a paradigm for NP drug delivery based on the principle that TAMs can sequester TNP payload and gradually release it into the surrounding tissue, thereby serving as ‘drug depots.’

## Results

### Dual imaging shows congruent vehicle and payload kinetics

**Figure 1:**
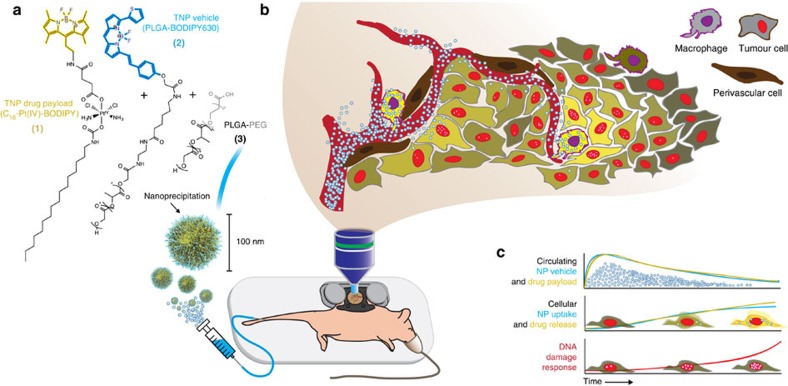
Overview schematic of intravital nanoparticle imaging for pharmacokinetic and pharmacodynamic analysis. Nanoparticles (**a**) were injected by tail-vein catheter and imaged in real time within the tumour using a dorsal window chamber (**b**). Imaging results enable simultaneous monitoring of circulating pharmacokinetics within the TMV (**c**, top); single-cell uptake of the nanoparticle vehicle and its drug payload (**c**, middle); and DNA damage response in tumour cells, monitored by 53BP1 puncta formation (**c**, bottom).

For clinical applicability and generalizability, TNPs in this study were designed to incorporate desirable properties of previously described polymeric nano-formulations that have entered clinical trials^5^, especially those of materials that have received Food and Drug Adminstration approval. TNPs were formulated by combining three compounds (compounds **1–3**, Fig. 1a) via nano-precipitation, using spectrally complementary derivatives of BODIPYs as ideal fluorophores for efficient nano-encapsulation that convey lipophilicity and robust *in vivo* imaging arising from *in vivo* structural stability, environmentally robust fluorescence, high brightness and high photostability^16^,^18^,^19^,^20^. Compound **3** (poly(D,L-lactic-*co*-glycolic acid)-*b*-poly(ethylene glycol); PLGA-*b*-PEG) self-assembles to form the hydrophobic PLGA core of the NP and hydrophilic PEG outer shell. For the cytotoxic payload, we used a novel Pt(IV) pro-drug (*c,c,t*-Pt(NH_3_)_2_Cl_2_-(hexadecylcarbamate)[4-((2-(4,4-difluoro-1,3,5,7-tetramethyl-4-dora-3a,4a-diaza-s-Indacene)ethyl)amino)-4-oxobutanoate]) with a C_16_ aliphatic carbon chain and a BODIPY (*λ*_ex_=498 nm, *λ*_em_=543 nm; Supplementary Fig. 1a) as the two lipophilic ligands axially coordinated to the Pt(IV) centre. NPs were similar in size (135±1 nm; standard error of the mean (s.e.m.), across *n*=12; Supplementary Fig. 1b-c), polydispersity (PDI=0.18±0.01; s.e.m. across *n*=12) and drug release rates (*t*_1/2 release_=15 h; *n*=2; Supplementary Fig. 1d) compared with previously published Pt(IV) nano-formulations^6^,^9^, and were stable in physiological saline (Supplementary Fig. 1e). Free carboxylic acid terminal groups on the PEG gave TNPs a slight negative charge (zeta-potential=−23±1.4 mV; s.e.m. across *n*=4), which has been shown in some cases to benefit tumoral accumulation^21^,^22^,^23^,^24^ and mirrors TNPs in the clinic^5^. C_16_-Pt(IV) remains relatively stable in whole blood^25^. However, upon reduction to the active Pt(II) compound cisplatin, dissociated BODIPY increases fluorescence by sevenfold owing to loss of quenching by Pt(IV) (Supplementary Fig. 1f-g). BODIPY fluorescence thus serves as an effective indicator of both pro-drug delivery and activation. Overall, these properties enable clearly distinguishable imaging of both the TNP vehicle and its payload, in a stable and clinically relevant nano-formulation platform.

We performed a series of *in vitro* experiments to characterize TNP behaviour in tumour cell culture (Supplementary Fig. 2a). Co-localization studies with fluorescent compartmentally localized proteins showed that the TNP vehicle moved from early to late endosomes over the course of 24 h (Supplementary Fig. 2b). Compared with the TNP vehicle, the TNP payload exhibited more diffuse intracellular localization, and co-localization peaked in the late endosome 24 h post treatment (Supplementary Fig. 2c). Payload fluorescence increased over time owing to Pt reduction and BODIPY de-quenching (Supplementary Fig. 2d). Dose–response TNP treatment indicated that intracellular fluorescence correlates well with overall Pt uptake (*R*^2^>0.99) and resulting DNA damage (Supplementary Fig. 3), thereby validating the use of fluorescence as a surrogate marker of cellular payload content. Together, these results provide evidence that, over the course of 24 h, TNPs accumulate in endosomes and lysosomal compartments, where the Pt pro-drug is activated through reduction to form cisplatin.

### TNPs exhibit long TMV half-life and perivascular cell uptake

**Figure 2:**
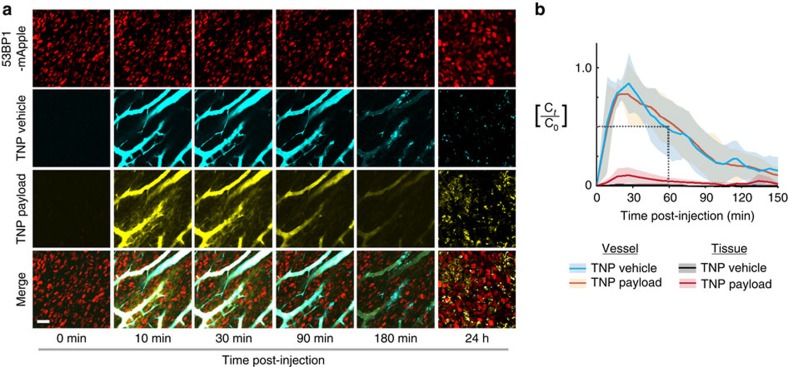
Pharmacokinetic analysis of nanoparticle shows extended microvasculature half-life and heterogeneous tissue accumulation. (**a**) Nanoparticle concentrations are monitored (**a**) and quantified (**b**) using time-lapse confocal fluorescence microscopy in the dorsal window chamber model. Nanoencapsulation extends the TMV half-life to 55 min, a >5-fold increase compared with unencapsulated Pt(II) compounds in the same animal model^29^. Scale bar, 50 μm. Thick lines and shading denote means±s.e.m. (*n*=6).

We first used time-lapse intravital imaging to characterize PK within a xenograft tumour model often used for intravital studies (Fig. 1)^16^,^26^,^27^,^28^. Tumours were generated from subcutaneously implanted HT1080 cells expressing a fluorescently tagged DNA damage response protein, 53BP1, which localizes to the nucleus^29^,^30^. TNPs reached the TMV within 10–15 min of intravenous injection, and at early time points (*t*<30 min) the vehicle and its payload exhibited strong co-localization within vessels (Fig. 2a; intravascular Pearson’s correlation ρ=0.8±0.02, s.e.m. across *n*=4; Mander’s M1 and M2>0.95). Both the vehicle and its payload showed initial vascular PK similar to each other (Fig. 2b; *t*_1/2 vehicle_=55±5 min; *t*_1/2 payload_=61±6 min; s.e.m. across *n*=6), to previous studies using similar nano-formulations entering the clinic^5^,^6^,^31^, and to clinical NP formulations such as PEGylated liposomal doxorubicin (DOXIL; *t*_1/2 initial_<5.2 h in humans and 2 h in rats^32^,^33^). Immediately as the TNPs reached the TMV, roughly 10% of the payload diffused into the surrounding tumour tissue. Such an initial burst of drug release was also observed *in vitro* (Supplementary Fig. 1d) and with similar previously described nano-formulations^34^,^35^, characterized by significantly faster release kinetics at initial phases followed by a later phase of slower release. Notably, PK imaging of BODIPY-labelled Pt compounds that were not nano-encapsulated revealed much more rapid vascular PK (*t*_1/2 unencapsulated_=10±5 min^29^). Therefore, despite an initial burst of Pt release seen both *in vitro* (Supplementary Fig. 1d) and *in vivo* (Fig. 2), nano-encapsulation conferred a relatively long circulation half-life.

Over the course of several hours, TNPs moved from the TMV (*t*<1 h) specifically to peritumoral host cells (1–3 h) adjacent to or even extending processes into the TMV. Uptake of TNPs by tumour cells themselves occurred more gradually and to a lesser degree compared with these perivascular host cells. By 24 h, TNPs were undetectable in vessels and had accumulated primarily in a heterogeneous mix of tumour cells and host cells, with host-cell uptake characterized by high local accumulation within cell-sized (7±1 μm; s.e.m. across *n*=4 tumours) diameters. At 24 h post-injection, the TNP payload also co-localized with the TNP vehicle in these host cells (Pearson’s correlation within tumour tissue: *ρ*=0.7±0.08; Mander’s M1 and M2>0.6±0.05; s.e.m. across *n*=4). Compared with the TNP vehicle, however, the payload accumulated more diffusely throughout the bulk tumour tissue, suggesting some payload release from the TNP vehicle (Supplementary Fig. 4a). We quantified these observations by measuring the coefficient of variation (CV) across cells within the tumour tissue (Supplementary Fig. 4b). The payloads exhibited >40% decrease in CV compared with the TNP vehicle, indicating more homogeneous distribution. In contrast to TNPs, naked BODIPY-labelled Pt that was injected in the absence of an encapsulating NP vehicle showed a substantially more homogeneous distribution within tumour tissue, with little preferential accumulation in any particular cell type (Supplementary Fig. 4; 60% reduction in CV). In summary, these data show that (i) nano-encapsulation enables a relatively long circulating half-life of the Pt(IV) pro-drug; (ii) nano-encapsulation influences spatial distribution within the bulk tumour tissue and (iii) TNPs primarily accumulate in perivascular host cells and, to a lesser extent, tumour cells.

### TNPs cause mitotic arrest and localized DNA damage

**Figure 3:**
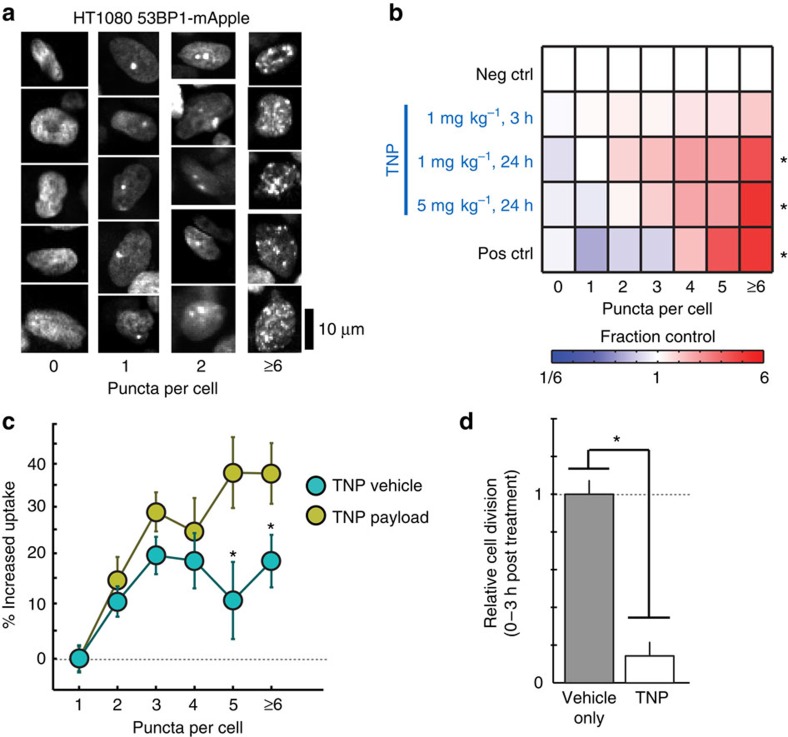
TNP causes mitotic arrest and DNA damage in a spatially defined dose-dependent manner. (**a**) Example images of 53BP1 puncta, 24 h post treatment with 1 mg kg^−1^ TNP, scale bar, 10 μm. (**b**) TNP treatment increases the fraction of cells exhibiting many 53BP1 puncta, measured across *n*≥3 animals and *n*>2,000 cells (*P<0.01, two-tailed *t*-test). Positive control (Pos. ctrl): 24 h supra-therapeutic cisplatin, 9 mg kg^−1^. (**c**) Heterogeneous local TNP vehicle and payload accumulation within 5 μm of single-cell nuclei correlate with corresponding DNA damage response in those cells (one-way analysis of variance; *n*>6,000 cells across *n*=5 animals; *P*<10^−21^ for both vehicle and payload); the local TNP payload correlates better with response compared with TNP vehicle (*P<0.05; two-tailed *t*-test). (**d**) Cell division events were quantified from time-lapse movies across *n*>1,000 cells (*P=0.001; two-tailed *t*-test; *n*=3 animals). All dosages in mg of Pt. Data are means±s.e.m. Neg ctrl, negative control.

**Figure 4:**
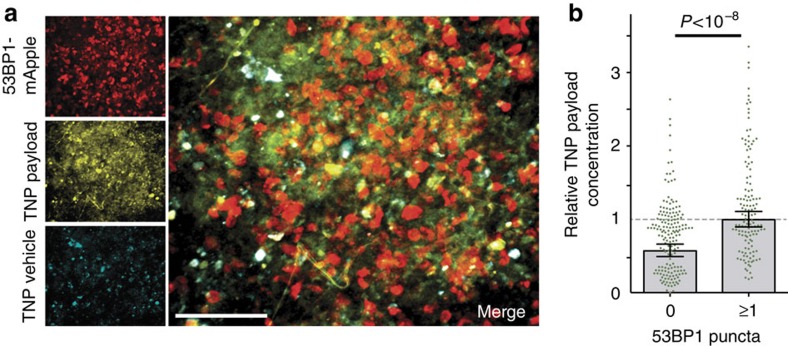
Local TNP payload accumulation correlates with DNA damage in OVCA. (**a**) Single-cell DNA damage response correlates with local TNP payload levels in a model of disseminated metastatic OVCA. A2780CP OVCA cells were injected intraperitoneally; 6 weeks later, TNP was i.v. injected, and the following day intraperitoneal lesions were excised and imaged for DNA damage response (using 53BP1 puncta) and TNP uptake. Scale bar, 50 μm. (**b**) OVCA cells exhibiting detectable DNA damage by 53BP1-puncta have higher TNP payload levels, compared with those without (two-tailed *t*-test; s.e.m. across *n*=3 animals).

In addition to PK analysis, high-resolution intravital imaging allowed for simultaneous monitoring of the cellular response to TNPs at the molecular level. Fluorescently tagged 53BP1 is a key member of the non-homologous end-joining pathway that forms visible puncta at sites of DNA double-strand breaks^29^,^36^. Based on the hypothesis that treatment with a cisplatin pro-drug would lead to increased DNA damage in tumour cells, we imaged 53BP1 puncta in response to TNP and quantitated the DNA damage response across populations of individual tumour cells at various time points (Fig. 3a). The results show a dose- and time-dependent increase in the fraction of tumour cells exhibiting high levels of DNA damage (Fig. 3b). We next tested whether there was an observable relationship between local payload accumulation and resulting single-cell DNA damage response. We used automated three-dimensional (3D) segmentation software to quantify TNP vehicle and payload accumulation within individual tumour nuclei, and correlated this information with DNA damage response in those cells. The results showed that cells exhibiting high levels of DNA damage also had a roughly 300% higher local payload concentration (Fig. 3c). Compared to TNP payload, local concentration of TNP vehicle also correlated with DNA damage, but to a lesser degree (Fig. 3c). DNA damage elicited by Pt treatment generally induces mitotic arrest *in vitro*^25^, but it has been difficult to directly observe this response *in vivo*, where anti-mitotics often behave much differently^26^. To determine TNP effects on cell growth, we used time-lapse intravital imaging to track individual tumour cells and mitotic events over the first 3 h following TNP injection in the HT1080 model (Supplementary Fig. 5) and found that TNP indeed caused a substantial reduction in tumour cell division (Fig. 3d). To test generalizability in another tumour model, we next examined local drug accumulation and DNA damage in an orthotopic model of disseminated ovarian cancer (OVCA)^37^. Human A2780CP OVCA cells were intraperitoneally injected in nu/nu mice, and 6 weeks later (once palpable tumours and/or ascites had developed), animals received TNP treatment. The following day, tumours were excised and imaged for TNP accumulation and DNA damage. Imaging shows similar TNP localization patterns as observed with the HT1080 model (Fig. 4a). We also found similar statistically significant correlation between local TNP payload levels and tumour cell DNA damage (Fig. 4b). Taken together, these results demonstrate that TNPs elicit substantial DNA damage response activity and corresponding growth arrest *in vivo.* In both the subcutaneous and orthotopic OVCA tumour models, the correlation between local drug accumulation and DNA damage response implies that NP-mediated drug delivery plays a significant role in governing such PD.

### Identification of cell populations with drug in steady state

**Figure 5:**
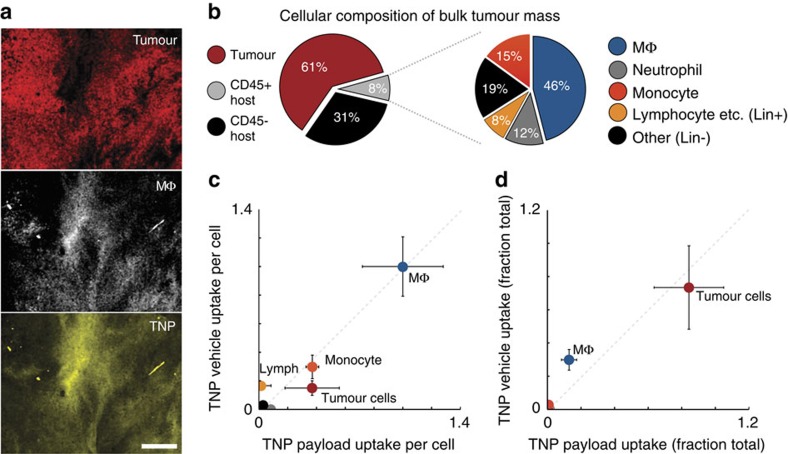
TNPs primarily accumulate in tumour cells and tumour-associated macrophages in steady state. (**a**) Intravital imaging of subcutaneous HT1080–53BP1-mApple tumours 24 h post injection with TNP (payload shown in yellow). TAMs (white) were labelled by dextran-coated nanoparticles injected 24 h prior. Scale bar, 100 μm. (**b**) Single-cell suspensions of the bulk tumour mass were immunostained and gated into various cell-populations, quantified here as fractions of the total number of cells analysed. (**c**) Macrophages accumulate the most TNP per cell. Using flow cytometry, uptake of both the TNP vehicle and its payload were quantified by fluorescence for each of the cell populations shown in **b**, 24 h post injection. CD45^−^ host cells, neutrophils and ‘other’ CD45^+^ leukocytes did not accumulate significant TNP vehicle or payload. Fluorescence values normalized to the average macrophage values, after subtracting background autofluorescence of each cell population measured from control-treated tumours. (**d**) Most TNP accumulates in tumour cells and macrophages within the bulk tumour mass. TNP uptake measurements from **c** were weighted against the relative prevalence of each cell population in **b** to calculate the total fraction of TNP uptake within each cell population. For **b**–**d**, error bars denote standard error (*n*≥4).

We next performed a combination of intravital imaging, flow cytometry and histology studies to determine in which immunologically defined cell populations the TNP vehicle and its payload accumulated within the bulk tumour mass at 24 h, after TNPs had largely cleared the circulation. With the same HT1080 xenograft model used in the previous imaging experiments, we labelled TAMs with a fluorescent dextran-coated NP^16^ and found significant TNP uptake in TAM-rich regions of the bulk tumour mass (Fig. 5a). For more detailed immunological examination, we next performed flow cytometry analysis of HT1080 tumours. Results show that tumour cells made up 61% of the bulk tumour; 31% of the cells within the bulk tumour were CD45^−^ host cells (erythrocytes, tumour associated fibroblasts, endothelium and others); and the remaining 8% were CD45^+^ leukocytes (Fig. 5b). TAMs made up the largest population of leukocytes within the bulk tumour and also accumulated the highest levels of both TNP vehicle and payload on a per-cell basis (Fig. 5c). Because tumour cells far outnumbered TAMs in the HT1080 xenograft model, the majority of TNP vehicle and payload within the bulk tumour still resided in tumour cells themselves (Fig. 5d). Nonetheless, even though TAMs comprised only 4% of cells within the total tumour mass, they accumulated more than 40% of the total injected TNPs relative to tumour cells, and 30% of the total TNP overall.

**Figure 6:**
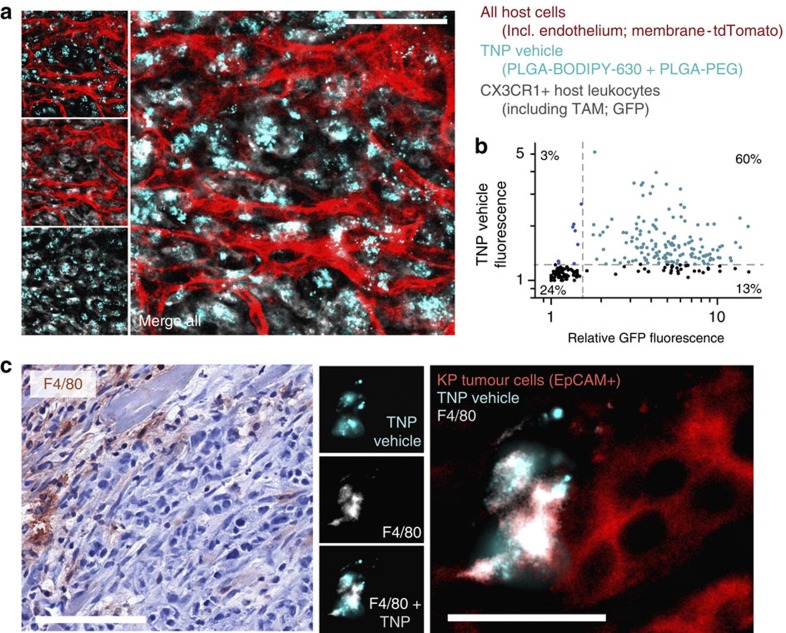
Tumour-associated CX3CR1^+^ and F4/80^+^ host phagocytes accumulate TNP vehicle in a syngeneic model of lung cancer. *Kras* mutant *p53*^−/−^ (KP) lung cancer cells were subcutaneously implanted into *Cx3cr1*^*GFP/+*^ reporter mice for directly visualizing GFP^+^ monocytes, dendritic cells and TAMs. Tumour-adjacent stromal regions were imaged 24 h following i.v. injection with TNP vehicle. Reporter mice expressed membrane-anchored tdTomato in all host cells, most visibly here with endothelium. Scale bar, 50 μm. (**b**) Fluorescence intensities of GFP and TNP vehicle were quantified across single-cells based on **a**, showing most stromal cells that accumulate TNP vehicle were also GFP^+^ (*n*=4 animals, *n*=250 cells). (**c**) Histological analysis of F4/80^+^ host phagocytes, 24 h post injection with TNP vehicle. Immunohistochemistry (left) shows tumours stained with haematoxylin and F4/80 (brown; scale bar, 100 μm), and corresponds with immunofluorescence (right) showing F4/80^+^ host phagocytes, EpCAM^+^ KP tumour cells, and local accumulation of TNP vehicle (scale bar, 25 μm). F4/80 labels a subset of GFP^+^ cells in *Cx3cr1*^*GFP/+*^ reporter mice^38^ (namely, macrophages).

To directly visualize genetically defined tumour-associated host leukocytes, we imaged TNP vehicle uptake in tumour-bearing fractalkine *Cx3cr1*^*GFP/+*^ reporter mice, which are known to contain GFP^+^ macrophages^38^. In this model, *KRAS* mutant *p53*^−/−^ (KP) cells derived from autochthonous lung tumours^39^ were subcutaneously implanted into immunocompetent *Cx3cr1*^*GFP/+*^ reporter mice, and TNP vehicle (without the green fluorescent payload) was intravenously administered. The vast majority of TNP vehicle accumulated within these GFP^+^ host leukocytes, especially near TMV (Fig. 6a,b). Using the above described KP lung cancer and intraperitoneal OVCA xenografts, we confirmed by histology that high levels of TNP vehicle accumulate in F4/80^+^ host phagocytes in these disease models as well (Fig. 6c and Supplementary Fig. 6). These analyses ultimately corroborate results from the intravital imaging data showing uptake of TNPs in tumour cells but also host cells, which are confirmed here as TAMs.

### TNP payload spatially redistributes from TAMs to tumour cells

**Figure 7:**
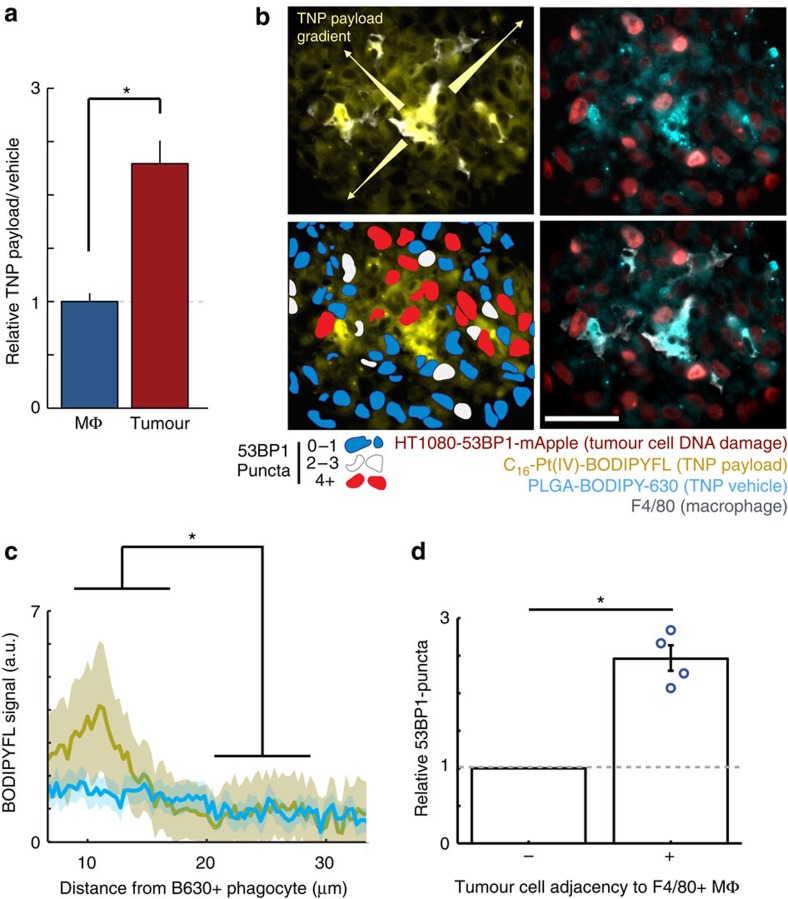
DNA-damaging TNP payload redistributes from TAMs to neighbouring tumour cells. (**a**) Compared with TAMs, tumour cells exhibit elevated TNP payload as a fraction of TNP vehicle (measured by flow-cytometry), suggesting payload redistribution (*P=0.005; two-tailed *t*-test; *n*≥4 per group). (**b**) Example immunofluorescence showing TNP vehicle uptake by F4/80^+^ macrophages and payload diffusion into surrounding tumour tissue. In lower left panel, red pseudo-colour indicates tumour cells with high levels of 53BP1 puncta. Of note, these cells are also adjacent to F4/80^+^ macrophages and contain elevated TNP payload. Scale bar, 50 μm. (**c**) By histological analysis, TNP payload but not vehicle is present at elevated levels within a 15-μm range of host phagocytes (*P=0.047, two-tailed *t*-test; *n*=3 animals). (**d**) By histological analysis, tumour cells immediately adjacent to F4/80^+^ macrophages exhibit elevated DNA damage response, compared with average DNA damage response seen in non-adjacent tumour cells for each of four animals shown by individual data points (*P=0.007; matched two-tailed *t*-test).

Based on intravital imaging evidence showing that the Pt(IV)-payload was more diffusely distributed within tumour tissue compared with its TNP vehicle (Supplementary Fig. 4), we next quantified the extent of payload redistribution and its effect on drug response. Using flow-cytometry data, we confirmed that tumour cells exhibited more than twice the amount of payload accumulation as a ratio of vehicle accumulation, compared with TAMs (Fig. 7a), thereby suggesting that payload may transfer from TAMs to tumour cells. We hypothesized that local payload release from TAMs into neighbouring tumour cells would lead to elevated payload concentration in these tumour cells, which would in turn enhance DNA damage. By histology we also measured greater than threefold elevated payload concentrations within roughly one cell-length (15 μm) of TAMs (Fig. 7b,c), and analysis of intravital imaging data provided further evidence that relevant payload concentration gradients occur over length-scales of<25 μm (Supplementary Fig. 7a). One explanation for these observations is that TAMs induce neighbouring tumour cells to take up more TNP directly; however, the TNP vehicle itself was not similarly elevated in the same neighbouring region (Fig. 7c). Tumour cells within the same phagocyte-neighbouring region also exhibited higher DNA damage on average, as determined by histology (Fig. 7d) and supported by corroborating intravital imaging analysis (Supplementary Fig. 7b). These analyses cumulatively show local payload redistribution from TAMs to neighbouring tumour cells exhibiting elevated DNA damage.

We next performed a series of *in vitro* experiments to directly test whether macrophages could release functional, DNA-damaging TNP payload into their surrounding environment. Similar to *in vivo* observations, we found that *in vitro* macrophages accumulated TNPs more rapidly and to a greater degree than tumour cells (Supplementary Fig. 8a). Once taken up by macrophages, Pt-payload was released at high levels into the extracellular supernatant. In fact, over the course of 24 h, more Pt was found in the supernatant than in the macrophages themselves (Supplementary Fig. 8b,c). The Pt-containing macrophage supernatant was both DNA damaging (Supplementary Fig. 8d) and cytotoxic (Supplementary Fig. 8e). In contrast, supernatant from macrophages treated with unencapsulated cisplatin at concentrations ranging from 1 to 300 μM was not cytotoxic to tumour cells (Supplementary Fig. 8e). For supernatant from TNP-treated macrophages, filtration with molecular-weight cutoff filters (3 and 100 kDa) did not reduce its potency, which indicated that cytotoxic effects were independent of macrophage-derived cytokines or other signalling proteins (Supplementary Fig. 8f). In addition, Pt-containing macrophage supernatant exhibited 50% inhibitory effect on tumour cell viability/cytotoxicity at Pt concentrations of 1.5±0.5 μM (s.e.m. across *n*=3) within the macrophage supernatant (Supplementary Fig. 7e), which is comparable to the potency of the Pt-payload when TNP was directly applied to tumour cells (IC_50_=1.7±0.02 μM; s.e.m. across *n*=2; Supplementary Table 1). Taken together, these combined *in vitro* and *in vivo* data further confirmed that TAMs can serve as drug depots that accumulate high levels of TNPs and then release their DNA-damaging cytotoxic payload to the surrounding tumour tissue.

### TAM depletion reduces TNP accumulation and efficacy

**Figure 8:**
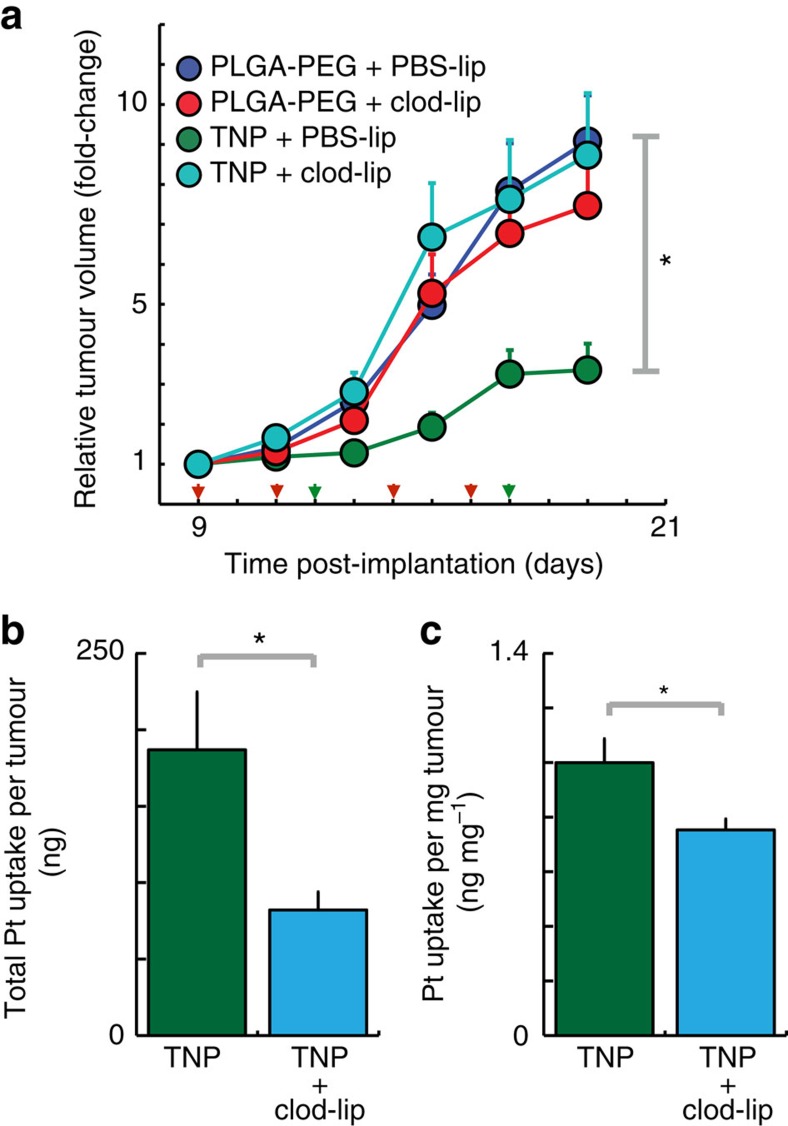
Macrophage depletion reduces intratumoral accumulation and efficacy of TNP. (**a**) TNP reduces tumour growth in a macrophage-dependent manner. Animals were treated with TNP (green arrows) and clodronate liposomes (clod-lip; red arrows) to systemically deplete macrophages. Drug-free PLGA-PEG polymeric NPs and phosphate buffered saline liposomes (PBS-lip) were used as vehicle controls. TNP significantly reduced tumour growth (*P*=0.039), whereas clod-lip did not (*P*=0.21); however, clod-lip significantly reduced the impact of TNP (*P*=0.039 interaction term; all *P*-values used the two-tailed two-way analysis of variance, total *n*=38 animals). (**b**,**c**) Macrophage depletion using liposomal clodronate reduced the total amount (**b**) and concentration (**c**) of intratumoral Pt, measured using AAS (*P*=0.02 and *P*=0.03, respectively, two-tailed *t*-test; total *n*=22).

Finally, we tested whether TAM depletion would reduce the therapeutic efficacy of TNP in cancers. Macrophages were systemically depleted by pre-treatment with clodronate liposomes (clod-lip)^40^ before TNP injection. In this tumour model, macrophage depletion itself had no detectable impact on tumour growth (*P*=0.2; two-tailed two-way analysis of variance; *n*=38 mice). However, it substantially reduced the degree to which TNP treatment was able to slow tumour growth (Fig. 8a). Direct measurement of intratumoral TNP accumulation confirmed that macrophages play a key role in mediating drug delivery: macrophage-depleted tumours exhibited both lower total intratumoral Pt (Fig. 8b) and a lower concentration of Pt (Fig. 8c) compared with macrophage-containing tumours, 24 h post administration of TNP, at which time no TNP remained circulating in the TMV of any tumour. The 24% decrease in Pt concentration (from 5 to 3.8 μM; Fig. 8c after converting to molarity) correlates well with the flow cytometry-based estimate that TAMs accumulate 30% of the total intratumoral TNP. It also correlates with the observation that unencapsulated Pt exhibits 37% lower intratumoral accumulation than nano-encapsulated Pt (Supplementary Table 1)^9^. This decrease disproportionately impacted TNP effects on tumour growth, probably because intratumoral Pt concentrations were near the threshold at which HT1080 tumour cells respond (*in vitro* IC_50_=3.7±0.1 μM for unencapsulated Pt(IV) payload; s.e.m. across *n*=2). TNP clearance kinetics, TMV leakiness and other EPR factors all may influence drug accumulation within the tumour; nonetheless, the experiments here show that all things being equal, macrophage uptake substantially contributes both to the delivery of TNP to the tumour and, consequently, to the therapeutic impact on tumour growth.

### Liver TNP uptake is well tolerated and blocks metastasis

Given our evidence for TAMs in mediating TNP delivery, we next investigated TNP effects in the liver, which is a prominent site of the mononuclear phagocyte system and is known to accumulate NPs^6^. Despite substantial liver accumulation (Supplementary Fig. 9a), TNPs did not significantly impact animal body weight (Supplementary Fig. 9b) and did not cause changes outside of the normal range in blood markers of renal or liver toxicity at the therapeutic dose (Supplementary Fig. 9c). Within the bone marrow, another important site of the mononuclear phagocyte system, TNP payload concentrations were much lower (Supplementary Fig. 9d,e). Although clinically relevant doses of un-encapsulated cisplatin caused a significant reduction in the number of nucleated cells in the bone marrow, TNP effects were not statistically significant (Supplementary Fig. 9f,g). Overall, these results demonstrate a positive safety profile despite liver accumulation.

**Figure 9:**
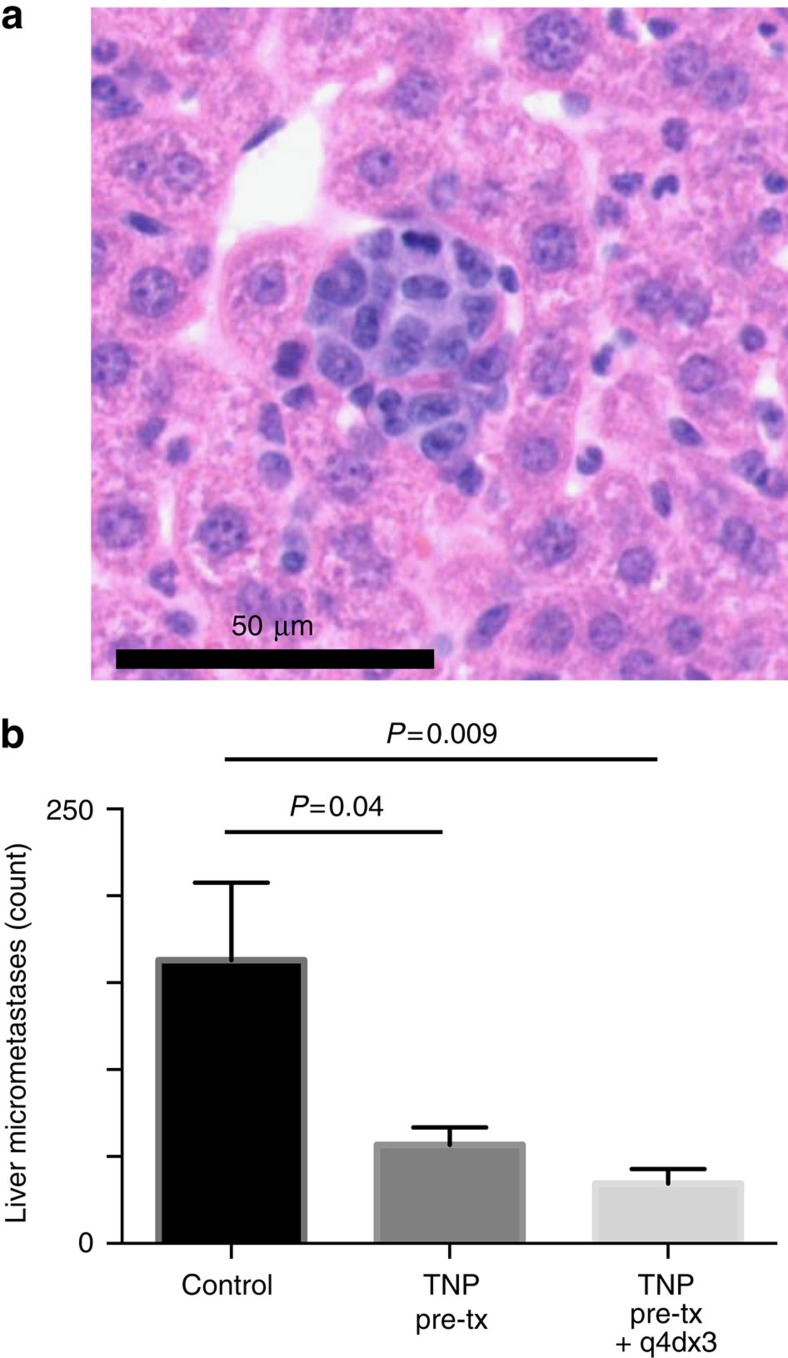
TNP delivery to the liver blocks development of micro-metastases. (**a**) Representative liver micro-metastasis, 2 weeks after i.v. injection of 4T1 breast cancer cells (haematoxylin and eosin staining of FFPE tissue section). (**b**) TNP reduces liver micro-metastasis development in the 4T1 model of haematogenous metastasis (two-tailed *t*-test; shown as mean±s.e.m.; *n*≥5). To initially deliver TNPs to host phagocytes (rather than tumour cells), TNPs were i.v. administered (1 mg kg^−1^) 6 h before i.v. injection of 4T1 cancer cells, such that TNP largely cleared the circulation at the time of cancer cell administration. One-third of the cohort received subsequent TNP treatments as indicated.

We next tested whether TNP treatment could block the development of liver micro-metastases in a model of haematogenous breast cancer dissemination. 4T1 murine breast cancer cells were intravenously injected into immunocompetent balb/c mice, thus forming tumours in the liver, lung and other organs. To primarily deliver TNPs to host phagocytes rather than tumour cells, we pre-treated naive mice with TNPs, waited 6 h for a majority of the drug to clear circulation, and then injected a single-cell suspension of 4T1 cells. Two weeks later, livers were excised and analysed by histology for tumour burden. Results show that pre-treatment with TNPs reduced the development of micro-metastases by over 65%, and repeated TNP doses caused a slightly greater reduction of 80% (Fig. 9). No treatment impacted tumour burden in the lung (two-tailed *t*-test; *n*≥5). Taken together, these results demonstrate that TNPs can safely accumulate in host cells of the liver, thus allowing TNP payload to block the development of local metastases from haematogenously disseminated cancer cells.

## Discussion

This work presents a new paradigm for employing TAMs as ‘drug depots’ for local delivery of nanotherapeutics to neighbouring tumour cells. Key to the development of this model was new technology for the *in vivo* visualization of multi-step drug delivery and the corresponding single-cell PD response at high spatiotemporal resolution. With this approach we demonstrated that nano-encapsulation allows for long circulating drug half-life and guides the delivery of drugs to TAMs, from which their functional cytotoxic payloads locally transfer to and damage neighbouring tumour cells. This paradigm most likely extends to other nano-encapsulated therapeutics and non-cancer disease indications in which macrophages accumulate near the target tissue. The clinically tested TNP platform used here^14^ can encapsulate other therapeutics including paclitaxel, doxorubicin and irinotecan, which exhibit release rates similar to the Pt(IV) payload^5^,^6^,^7^. Of relevance is that local macrophage accumulation occurs in multiple diseases including type 1 diabetes^41^, rheumatoid arthritis^42^, endometriosis^43^ and cardiovascular diseases such as atherosclerosis and myocardial infarction^44^,^45^. The imaging approach we describe may thus apply to these other pathologies, especially where intravital microscopy has already been successfully used^46^. Although macrophages themselves are often pharmacological targets^47^, concomitant drug delivery to surrounding tissue may be advantageous^48^. Overall, the drug delivery paradigm presented here, along with the complementary imaging technology, portend broad applicability to other TNP strategies and disease indications.

TAMs have been extensively studied and targeted for their roles in disease development, progression and therapeutic response^48^,^49^. Nonetheless, TAMs have received less consideration in the context of novel nanotherapeutics entering the clinic. Because of their secretion of growth factors, cytokines and proteases, TAMs have been implicated in supporting disease progression by enhancing angiogenesis, metastasis and cancer stem cell niche formation, all while suppressing anti-cancer immune responses. In multiple indications, high TAM accumulation at tumour sites correlates with poor prognosis^50^,^51^. Although TAM-mediated tumour neo-vascularization can increase vascular leakage and corresponding therapeutic delivery^52^, TAMs also provide chemoprotective cues to tumour cells, and TAM infiltration following chemotherapy correlates with poor treatment response^52^,^53^. For these reasons, therapeutic TAM killing or TAM re-polarization into an anti-cancer phenotype represents an active area of development, for example, by targeting colony-stimulating factor 1 receptor (CSF1R)^54^,^55^,^56^. In several examples, blocking leukocyte infiltration via CSF1R inhibition or chemokine receptor type 2 ablation enhances response to chemotherapeutics including cisplatin^52^. However, very little experimental data describes how TAM depletion affects the response to nanotherapeutic formulations, and evidence presented here suggests that complete TAM elimination would be undesirable in such contexts.

In contrast to previous reports that focus chiefly on traditional unencapsulated cytotoxic therapies, we show here that TAMs are critical mediators of drug action for nano-encapsulated therapeutics. TAMs and other leukocytes are implicated in taking up a wide range of clinically relevant nanotherapeutics, including liposomal^57^, polymeric^58^ and albumin-binding^59^ formulations. Nonetheless, in the past, imaging capabilities have generally not allowed detailed insight into the kinetics of drug uptake, payload redistribution and corresponding PD responses at the single-cell or subcellular level to be determined for these compounds^57^,^60^. Compared with polymeric NPs such as those used here, liposomal formulations generally are associated with slower payload release and decreased payload bioavailability, and there is little evidence that doxorubicin is released from TAMs after they have taken up liposomes^57^,^61^. In fact, previous failures to develop liposomal cisplatin have been attributed to insufficiently fast drug release^13^. By contrast, polymeric nano-formulations can be designed to release payloads at prescribed rates^5^ and even at programmed environmental (for example, pH) conditions^62^. Here, TNPs were engineered to release their payload over a slightly longer time scale (50% drug release at 15 h; Supplementary Fig. 1d) relative to the observed PKs of cellular uptake (TAM uptake observable at 1 h and gradually increases by 24 h), and therefore were ideally suited for TAM-mediated drug delivery as described herein. Overall, this work builds on previous studies that implicate macrophages in TNP uptake; however, through engineered drug release and fluorescence imaging strategies, we demonstrate how the TNP payload can redistribute to neighbouring tumour cells at the site of local TAM accumulation in a therapeutically beneficial manner.

The results presented have implications for future development and clinical use of TNPs. Given that TNPs accumulate in TAMs at high levels, it follows that one criterion for selecting patients into TNP trials may be the degree of peritumoral TAM content, which can be assessed by magnetic resonance imaging using clinically approved NP contrast agents such as ferumoxytol (Feraheme)^63^ or experimental theranostics including drug-conjugated ferumoxytol derivatives^64^,^65^. TAM content is especially important in the context of therapies that either increase or decrease infiltration of macrophages to tumours. For instance, doxorubicin^52^ and CD40 agonist antibodies^66^ enhance macrophage infiltration, whereas CSF1R inhibitors^56^ and the clinically approved trabectedin^67^ decrease TAM accumulation. The paradigm of TAM-mediated drug delivery presented offers a compelling case for consideration of competing drug effects on TAM recruitment, and highlights the potential use of TNP vehicles for more efficient (via improved kinetics and/or targeting subsets) targeting of both TAMs and their neighbouring tumour cells.

## Methods

### C_16_-Pt(IV)-BODIPY synthesis

A 0.2-ml N,N-Dimethylformamide (DMF) solution of *cis,cis,trans-*[Pt(NH_3_)_2_Cl_2_(O_2_CCH_2_CH_2_COOH)(OCONH(CH_2_)_15_CH_3_)] (15 mg, 21 μmol) and 1-[Bis(dimethylamino)methylene]-1H-1,2,3-triazolo[4,5-b]pyridinium 3-oxid hexafluorophosphate (HATU) (8 mg, 21 μmol) was stirred at room temperature (RT) for 15 min. To the clear solution was added a 0.4 ml DMF suspension of the amino BODIPY (4,4-difluoro-5,7-di- methyl-4-bora-3a,4a-daiza-s-indacene; 6 mg, 21 μmol), and the orange suspension was stirred at RT for 30 min. Finally, a 4 μl portion of N,N-Diisopropylethylamine (DIPEA) was added to the mixture and the resulting orange solution was stirred at RT for 5 h. A 5-ml volume of saturated aqueous NaCl was added to the reaction mixture and a red precipitate was obtained. The precipitate was washed with 3 × 3 ml water. The resulting orange solid was dried in the vacuum overnight and the product was isolated by silica chromatography (acetone, *R*_f_=0.5). Yield: 7 mg (30%). ^1^H NMR (400 MHz, acetone-d_6_): *δ*: 7.73 (s, 1H, NHCH_2_CH_2_-BODIPY), 6.54 (m, 6H, NH3), 6.18 (s, 1H, BODIPY), 5.80 (s, 1H, NH_carbamate_), 3.42 (m, 2H, NHCH_2_CH_2_-BODIPY), 3.29 (m, 2H, NHCH_2_CH_2_-BODIPY), 3.01 (m, 2H, CH_2_(CH_2_)_14_CH_3_), 2.45 (s, 6H, BODIPY), 2.21 (m, 10H, BODIPY, succinate), 1.20 (m, 28H, CH_2_(CH_2_)_14_CH_3_), 0.8(t, 3H, CH_2_(CH_2_)_14_CH_3_). ^13^C NMR (100 MHz, DMSO-d_6_): *δ*: 180.4, 172.1, 164.4, 153.9, 143.0, 142.0, 131.5, 122.3, 41.5, 40.9, 31.9, 31.8, 30.3, 29.5, 29.4, 29.2, 29.1, 22.6, 16.5, 14.6, 14.4. ^195^Pt NMR (86 MHz, DMSO-d_6_): *δ*: 1,240.1. High resolution electrospray ionization mass spectrometry (HR-ESI-MS) (positive mode) for C_36_H_63_BCl_2_F_2_N_6_O_5_Pt: *m*/*z* [M−H]^+^ calculated: 975.4020; observed: 975.4001.

### Polymer materials

Unless otherwise stated, all chemical reagents were purchased from Sigma-Aldrich. PLGA(55:45 lactide/glycolide)_35.2kDa_-PEG_3.5 kDa_ was purchased from Advanced Polymer Materials, Inc. (Cat. #13–01-CX-55/45–35.2k/3.5k; lot 14–02–169). From the vender, gel permeation chromatography indicated *M*_n_=38.8 kDa, *M*_w_=58.8 kDa and PI=1.52. PEG weight fraction (8.7%±0.3%) and molecular weight were confirmed by ^1^H NMR peak integration values, which were also used to calculate the lactide/glycolide ratio. ^1^H NMR in CDCl_3_: *δ* (in p.p.m.) 5.12–5.28 (m, (-OC**H**(CH_3_)CO)), 4.66–4.9 (m, (-OC**H**_**2**_COO-)), 3.64 (s, -OC**H**_**2**_CH_2_O-), 1.52–1.62 (m, (-OCH(C**H**_**3**_)CO-). Differential scanning calorimetry was performed (Q10; TA Instruments), heating from −60 °C to 100 °C at 10 °C min^−1^, with no apparent crystallization or melting temperature (Tm); glass temperature (Tg) was observed at 39.5 °C. BODIPY-630-NH_2_ (Life Technologies; 2.8 mg, 0.0046, mmol) was conjugated to PLGA(50:50 lactide:glycolide)_30–60 kDa_ (100 mg, ca 0.0023, mmol; Sigma) using previously published procedures^27^, by stirring at RT under Ar for 10 min, adding 1-Ethyl-3-(3-dimethylaminopropyl)carbodiimide (EDCI) (4.4 mg, 0.023 mmol) and 4-Dimethylaminopyridine (DMAP) (2.8 mg, 0.023 mmol) in CH_2_Cl_2_ (0.5 ml), and stirring overnight. Polymer was precipitated in cold 1:1 MeOH/Et_2_O, centrifuged (2,700*g*, 10 min) and repeatedly washed in minimal CH_2_Cl_2_, followed by MeOH/Et_2_O precipitation and centrifugation. The resulting blue precipitate was dried under vacuum.

### NP synthesis and characterization

TNPs were prepared by nano-precipitation. Briefly, the following reagents were dissolved in 50:50 (v/v) DMF/acetonitrile (ACN) at the indicated concentrations: PLGA-BODIPY-630 (0.17 mg ml^−1^), C_16_-Pt(IV)-BODIPY (0.17 mg ml^−1^) and PLGA-PEG (0.83 mg ml^−1^). The resulting solution was then mixed and added drop-wise to nuclease-free H_2_O while maintaining a ratio of 20:1 H_2_O to organic solvent. TNPs were stirred overnight and filtered through sterile 0.45 μm syringe filters (regenerated cellulose, 17 mm, Cole Palmer). The TNPs were concentrated by centrifugation using centrifugal filter units (Amicon; Millipore; molecular weight cut-off, MWCO=100 kDa). The concentrated TNPs were washed twice with de-ionized H_2_O and re-suspended in 1 ml of nuclease-free H_2_O. TNPs were diluted ten times in H_2_O and characterized by size using dynamic light scattering (DLS; Malvern Zetasizer). Zeta-potential was also measured by DLS in dI-H_2_O (−25±2.5 mV) and PBS, pH 7.4 (−23±1.4 mV). Although the buffered solution produced a slightly higher zeta-potential, measurements were not statistically different from each other (two-tailed *t*-test; *α*=0.05). The Pt concentration was determined by graphite furnace flameless atomic absorption spectroscopy (AAS). BODIPY-630 concentration was determined by a microplate fluorimeter after diluting TNPs in DMF and using pure BODIPY-630 for a standard curve. Transmission electron microscopy (TEM) experiments were performed on a JEOL 1011 electron microscope (JEOL). The TEM sample was prepared by depositing 10 μl of TNPs (5 mg ml^−1^) onto a carbon-coated copper grid. The excess solution was blotted, and the grids were immersed in a solution of phosphotungstic acid stain. The stain was blotted, and the dried grids were immediately used for imaging. To characterize payload release from TNPs (Supplementary Fig. 1d), they were incubated in PBS at 37 °C, filtered using 100 kDa MWCO centrifugal filters (Amicon; Millipore) at the indicated time points and resuspended in fresh PBS. Filtrate was frozen for later analysis by AAS. After 72 h, both the TNPs and the filtrate were analysed by AAS to quantify the remaining Pt. Based on the above procedure, <5% of total PLGA-BODIPY-630 was released from TNPs over the course of 48 h.

### Animal models

All animal research was performed in accordance with guidelines from the Institutional Subcommittee on Research Animal Care (Massachusetts General Hospital). All experiments were performed using female mice that were 5- to 7-week old at the start of the experiment. For experiments with HT1080 tumours, 2 million cells in PBS were subcutaneously implanted into nu/nu mice; roughly 2–3 weeks later (once tumours reached ∼8 mm diameter), imaging experiments were initiated. For the experimental model of haematogenous metastatic dissemination, 0.25 million 4T1 breast cancer cells suspended in PBS were intravenously (i.v.) injected into balb/c mice; roughly 2 weeks later tumours were excised for histological quantification of liver and lung tumour burden in formalin-fixed paraffin-embedded (FFPE), haematoxylin and eosin-stained sections. For intraperitoneal OVCA imaging, 10 million A2780CP cells suspended in PBS were intraperitoneally injected into nu/nu mice. TNP injection and imaging were performed once ascites or tumour masses became evident (roughly 6 weeks later). For histology and imaging experiments using KP cells, 1 million cells were subcutaneously implanted into C57Bl/6 background animals (all JAX), including *Cx3cr1*^*GFP/+*^*R26*^*mT*−*mG/+*^ dual-reporter mice. For dual reporter mice generation, *Cx3cr1*^*GFP/GFP*^ animals were crossed with *R26*^*mT*−*mG/mT*−*mG*^ animals that ubiquitously express membrane-anchored tdTomato^68^. The resulting *Cx3cr1*^*GFP/+*^ *R26*^*mT*−*mG/+*^ animals can be used to visualize GFP^+^ tdTomato^+^ macrophages, monocytes and dendritic cells, as well as tdTomato^+^ stroma and endothelium.

### Cell lines

With some exceptions, cell lines were obtained from American Type Culture Collection and routinely cultured according to the manufacturer’s guidelines. OVCA-429 were a kind gift from Dr Michael Birrer (MGH), and A2780CP were from Sigma. The murine lung adenocarcinoma cell line KP1.9 was generated from lung tumour cells of Kras^LSL−G12D/+^;p53^fl/fl^ mice (C57/Bl6 background), which develop lung adenocarcinoma after infection with an adenovirus expressing Cre recombinase by intratracheal administration^39^. KP cells were maintained in Iscove’s DMEM media supplemented with 10% fetal bovine serum and 5% penicillin/streptomycin. All cell lines were routinely tested for contamination using mouse antibody production testing (VRL Laboratories) and assaying for mycoplasma (VRL Laboratories; Lonza MycoAlert). The fluorescent protein mApple was subcloned into the pLVX vector using restriction enzymes *Afe*I and *Not*I. We inserted a *Xho*I site at in the 5′ end of the pLVX-mApple multiple cloning site using QuikChange site-directed mutagenesis (Agilent). The pDsRed-Monomer-Mem membrane targeting sequence (Clontech) was PCR amplified for In-Fusion cloning to construct pLVX-Mem-mApple. PCR product was then ligated into pLVX-Apple after *Afe*I and *Xho*I digestion, and fully sequenced. Lentiviral particles were produced using Lenti-X HTX Packaging System (Clontech) for transduction into HT1080 and A2780CP cells, which were then selected using 3 μg ml^−1^ puromycin. HT1080–53BP1-mApple and A2780CP-53BP1-mApple were developed as previously described^29^.

### Intravital microscopic imaging

TNPs were injected at the indicated dose (1 mg kg^−1^ unless otherwise stated) via tail-vein catheter immediately after mixing to a final 1 × PBS solution, at a final volume of 100 μl. Intravital microscopy was performed on an Olympus FV1000 multiphoton imaging system using a XLUMPLFLN × 20 water immersion objective (numerical aperture=1.0; Olympus America). Images were scanned sequentially using 405-, 473-, 559- and 633-nm diode lasers in combination with a DM405/488/559/635-nm dichroic beam splitter. Emitted light was then separated and collected using appropriate combinations of beam splitters (SDM473, SDM560 and/or SDM 640) and emission filters BA490–540, BA575–620, BA575–675 and/or BA655–755 (all Olympus America). Dextran pacific blue (*λ*_ex_=405 nm) was injected to initially image TMV as previously described^29^. Briefly, 500-kDa amino-dextran (Thermo) was labelled with Pacific Blue succinimidyl ester (Thermo), purified using 30 kDa MWCO centrifugal filtration (Amicon), and 250 μg i.v. injected 10 min before imaging. Dorsal window chamber imaging was performed following previously described procedures^29^,^69^,^70^. Briefly, 2 million HT1080–53BP1-mApple cells were suspended in 50 μl PBS and injected under the fascia of nu/nu mice (Cox7, MGH) 30 min following surgical chamber implantation, and imaged 2 weeks later.

### Histology

Excised tumours were embedded in optimal cutting temperature (OCT) compound, flash-frozen, sectioned using a Leica CM1900 Rapid Sectioning Cryostat (Leica), stained using anti-mouse F4/80 antigen eFluor 450 (clone BM8; eBioscience), and imaged using an upright Olympus BX63 microscope and a × 100 oil-immersion objective. For immunohistochemistry, a biotinylated anti-rat IgG antibody was applied, and VECTASTAIN ABC kit (Vector Laboratories Inc.) along with a 3-amino-9-ethylcarbazole substrate (Dako) were used for colour development. The sections were counterstained with Harris haematoxylin solution (Sigma-Aldrich) and scanned by using Nanozoomer 2.0RS (Hamamatsu). For immunofluorescence, anti-rat IgG-Alexa Fluor 405 (Abcam) as a secondary antibody and anti-mouse CD326 (EpCAM)-PE antibody (clone: G8.8, eBioscience) were used.

### *In vitro* NP characterization

For cytotoxicity assays (Supplementary Table 1), 5,000 cells per well were plated in 96-well plates, treated the next day with Pt-compound or the appropriate buffer control (DMF or drug-free PLGA-PEG NPs, as appropriate), and assessed for viability 72 h later using PrestoBlue (Life Technologies) following the manufacturer’s protocol. IC_50_ values for each compound were calculated by interpolating from an 11-point dose–response curve.

For live-cell *in vitro* microscopy of TNP uptake (Supplementary Fig. 2a), HT1080–53BP1-mApple cells were seeded on an optical-bottom 96-well plate (Ibidi; Applied Biophysics), treated the following day with 1 μM TNP for 24 h, washed in warm medium, and immediately imaged on a DeltaVision (Applied Precision) modified Olympus BX63 microscopy system with an environmental chamber. Dose–response analysis of TNP uptake (Supplementary Fig. 3) followed the same protocol. Corresponding measurements of intracellular Pt uptake (Supplementary Fig. 3d) were performed by treating 80% confluent cells in six-well plates for 24 h with TNP, rinsing with PBS, trypsinizing, centrifuging the cell pellet for 300*g* for 5 min, re-suspending the pellet in 50 μl fresh PBS, and freezing it for subsequent AAS analysis.

CellLight BacMam (Life Technologies) fluorescent protein-signal peptide fusions, delivered using baculovirus, were used for subcellular compartment co-localization experiments (Supplementary Fig. 2b). Briefly, 10,000 HT1080 cells were treated overnight with 20 μl CellLight reagent in 12-well dishes, plated the next day on collagen-I-coated glass coverslips in six-well dishes, and the following day were treated with 0.5 μM TNP for either 3 or 24 h. Following treatment, cells were counterstained with Hoechst 33342 and immediately imaged. Collagen-coating was performed by treating coverslips with 100 μg ml^−1^ rat-tail collagen-I (BD Biosciences) overnight and then rinsing with PBS.

To analyse de-quenching of the C_16_-Pt(IV)-BODIPY compound (Supplementary Fig. 2d), TNPs were added at 1 μM to full serum medium (DMEM+10% fetal bovine serum+1% penicillin/streptomycin) in the presence or absence of 90% confluent HT1080 in 96-well plates. Fluorescence was monitored by a Tecan Safire 2 fluorescence plate reader. For monitoring TNP uptake kinetics (Supplementary Fig. 8a), equal numbers of RAW 264.7 macrophages or HT1080 cells were plated in 96-well plates and incubated with 1 μM TNP. At the indicated time points, medium was temporarily transferred to empty wells and replaced with fresh media on cells. Cells were then immediately measured using a plate reader for BODIPY-630 fluorescence, and the TNP-containing media were replaced onto the cells again.

To analyse Pt uptake and release into supernatant by macrophages (Supplementary Fig. 8c), confluent 10 cm tissue-culture dishes of RAW macrophages were treated with 62 μM TNP for 24 h, rinsed in PBS, treated with fresh media, incubated for another 24 h and then analysed for Pt content by AAS. The supernatant was clarified by centrifugation (300*g* for 5 min) before AAS analysis. Cells were harvested by trypsinization, centrifugation (300*g* for 5 min), resuspension of the cell pellet in 50 μl PBS and freezing for subsequent AAS analysis.

To analyse whether the Pt-containing macrophage supernatant could invoke a DNA damage response in tumour cells (Supplementary Fig. 8d), RAW macrophages were treated for 6 h with 15 μM TNP, then rinsed and incubated in fresh media. After 24 h, media were clarified by centrifugation (300*g* for 5 min), transferred to HT1080–53BP1-mApple cells plated on optical-bottom 96-well plates (Ibidi; Applied Biophysics), and incubated overnight. The following day cells were rinsed and immediately imaged.

To analyse whether Pt-containing macrophage supernatant cause cytotoxic effects on tumour cells (Supplementary Fig. 8e), RAW macrophages were treated for 24 h with either TNP or unencapsulated C_16_-Pt(IV)-BODIPY (the latter to achieve high Pt concentrations), then rinsed and incubated in fresh media. After 24 h, media were clarified by centrifugation (300*g* for 5 min), then transferred to 5000 HT1080 cells per well of a 96-well plate. After 72 h, the cell count was determined by the PrestoBlue assay (Life Technologies).

To analyse whether the above cytotoxic effects depend on proteins in the Pt-containing macrophage supernatant (Supplementary Fig. 8f), RAW macrophages were treated for 16 h with 60 μM TNP, then rinsed and incubated in fresh media. After an additional 16 h, media were clarified by centrifugation (300*g* for 5 min), filtered using MWCO centrifugal filters (Amicon; Millipore), and transferred to 5000 HT1080 cells per well of a 96-well plate. After an additional 72 h, the cell count was determined by the PrestoBlue assay (Life Technologies).

### Tumour growth

Subcutaneous tumours were implanted in 7-week-old female nu/nu mice (Cox7, MGH), injected in four quadrant animal flanks as 1 million HT1080 cells suspended in 50 μl PBS. Nine days later, animals were ranked and sorted into groups of four based on tumour size. According to predefined protocol guidelines, mice were killed when tumour burden reached more than 1 cm in diameter, or 2 cm in diameter if only one tumour was present. Tumour volumes were measured two to three times per week, estimated using the spherical tumour volume formula *V*=^4^/_3_ *πr*^3^, where *r* is averaged from four caliper measurements performed by two blinded researchers. Each size-group was randomly distributed to the four treatment-groups such that final treatment-groups exhibited roughly equal distribution in tumour size, with 9–10 mice per group. Groups received 150 μl of clodronate liposomes (clod-lip) or PBS liposomes as a vehicle control, intraperitoneally, on the indicated days (Fig. 8a). Liposomes were from ClodLip BV (Haarlem), with clodronate at a concentration of 5 mg ml^−1^. As separate intravenous injections on indicated days (Fig. 8a), groups received 1 mg kg^−1^ Pt of either TNP or the TNP without Pt(IV) as a vehicle control. TNPs for this experiment were formulated as described above, using PLGA_8kDa_-PEG_5kDa_ and non-fluorescent PLGA_30–60kDa_ rather than PLGA_30–60kDa_-BODIPY-630. TNP formulations were made fresh before each injection, and batches were tested for size and monodispersity by DLS and TEM. Pt loading was measured by AAS, and appropriate drug release was measured as previously described. At 19 days post implantation, tumours were excised, rinsed in PBS and processed for histology by freezing in OCT compound, or alternatively processed for AAS analysis by blotting tissue to remove excess water and freezing. One of thirty-eight animals was prematurely killed owing to biting and cachexia.

### Flow cytometry

Subcutaneous tumours (HT1080 cells) were harvested from nu/nu mice 3 weeks post implantation, cut into small pieces and incubated in RPMI 1640 medium containing 0.2 mg ml^−1^ collagenase type I (Worthington Biochemical Corporation) for 1 h at 37 °C while shaking (250 r.p.m.). Digested tumours were filtered through a 70-μm cell strainer (BD Falcon). The resulting single-cell suspensions were washed and resuspended in PBS with 0.5% BSA and 2 mM EDTA. Cell labelling was performed with appropriate antibodies as indicated below for 45 min at 4 °C. The following cell types were identified by flow cytometry (LSRII, BD Biosciences) depending on cell marker expression: tumour cells (hCD29^+^), macrophages (CD45^+^CD11b^+^Ly6C^−^Lin^−^CD11c^+^F4/80^+^), neutrophils (CD45^+^CD11b^+^Ly6C^+^Lin^+^) and lymphocyte-like cells (CD45^+^Lin^+^CD11b^−^Ly6G^−^). The anti-human CD29 antibody (clone MAR4) was from BD, and the following anti-mouse antibodies used were from BioLegend: CD45 (clone 30-F11), F4/80 (clone BM8), CD11c (clone N418), Ly6C (clone HK1.4). CD11b (clone M1/70) was from BD Biosciences, along with all antibodies used in the lineage (Lin) mixture: anti-CD90.2 (clone 53–2.1), anti-B220 (clone RA3–6B2), anti-NK1.1 (clone PK136), anti-CD49b (clone DX5), anti-Ter119 (cloneTER-119) and anti-Ly6G (clone 1A8). To exclude dead cells, 7-aminoactinomycin D (Sigma-Aldrich) was used. Data were analysed using FlowJo v.8.8.7 (Tree Star, Inc.) and MATLAB. BODIPY-630 and amino-BODIPY fluorescence was assessed by using an LSRII flow cytometer and the mean fluorescence intensity (MFI) of the different cell populations was determined. Background autofluorescence for each immunologically defined cell population was measured from control-treated animals that were injected with non-fluorescent PLGA-PEG vehicle; this value was subtracted from the data for the fluorescent TNP-treated animals.

### Computational image analysis

Intravital microscopy images were analysed using either Matlab (Mathworks) or ImageJ and were pre-processed using background subtraction based on data acquired immediately before TNP injection. For vascular half-life calculations, fluorescence time-lapse values from multiple vessels across multiple animals were recorded, averaged and fit to an exponential decay model using nonlinear regression in MATLAB. Single-cell 3D segmentation for image cytometry analysis was performed using a custom MATLAB pipeline. Briefly, a previously described 3D nuclear-segmentation algorithm was modified to measure the MFI of TNP vehicle and its payload within a given radius of each nucleus. These measurements were then correlated with observed 53BP1-puncta in each individual nucleus, validated by eye (Fig. 3c). Throughout the manuscript, data falling >2 standard deviations from the mean were excluded as outliers from the statistical calculations. For calculation of spatial heterogeneity, measured by the CV (Supplementary Fig. 4), cell-sized regions of interest within images were quantified for MFI. CV for TNP and unencapsulated Pt (CP-11 (ref. 29)) were measured >20 half-lives post injection as an approximation of steady-state tissue distribution (5 and 24 h, respectively).

### Liver and BM analysis

Pt concentrations in the liver and bone marrow were determined by AAS and fluorescence imaging. Briefly, naive female nu/nu mice 8-week old were i.v. injected with TNP at a dose of 1 mg kg^−1^ Pt. 24 h later, animals were perfused with PBS by intracardiac injection before dissection. For fluorescence quantification of TNP payload, livers were excised and frozen in OCT compound for later sectioning. Femoral BM was flushed using PBS and smeared on a glass slide for immediate imaging. Un-injected control animals were used to measure background signal for each organ, which was baseline-subtracted in the quantification. For AAS analysis of Pt content, livers were excised, patted dry and frozen. BM was flushed using PBS, centrifuged at 16,000*g* to pellet cells and frozen. Tissue was then dissolved in 70% nitric acid and analysed as with the HT1080 tumours (Fig. 8). For quantification of BM cellularity, femurs were excised 24 h post injection with TNP, formalin-fixed overnight, decalcified in EDTA and processed for FFPE sectioning. Immunohistochemistry was performed for apoptosis analysis by terminal deoxynucleotidyl transferase dUTP nick-end labelling, using Apoptag Peroxidase In Situ Apoptosis Detection Kit (Millipore), counterstaining with Harris haematoxylin solution (Sigma-Aldrich), and scanning with a Nanozoomer 2.0RS (Hamamatsu).

Metastasis burden in the lung and liver was quantified by morphological assessment in haematoxylin and eosin-stained liver FFPE sections that had been imaged using Nanozoomer 2.0RS, with organ excision at time of animal euthanasia according to the institutional guidelines (body condition score of 2) or 20 days post cancer injection, whichever came first. No statistically significant difference was observed in animal survival, body weight loss or lung tumour burden across the groups. One mouse from the group receiving repeated TNP dosing was excluded from analysis since no lung tumours (and very few liver tumours) were found by histology, presumably due to failed initial intravenous injection.

Clinical chemistry measurements in Swiss albino mice were performed using previously described TNP and methods^6^. Graphically re-plotted here for context (Supplementary Fig. 9a), bio-distribution of Pt in male rats was measured as described previously^6^.

## Additional information

**How to cite this article:** Miller, M. A. *et al.* Tumour-associated macrophages act as a slow-release reservoir of nano-therapeutic Pt(IV) pro-drug. *Nat. Commun.* 6:8692 doi: 10.1038/ncomms9692 (2015).

## Supplementary Material

Supplementary Information Supplementary Figures 1-9, Supplementary Table 1 and Supplementary References
