# Supplementary material for: Tumour-associated macrophages act as a slow-release reservoir of nano-therapeutic Pt(IV) pro-drug
Source: Nat Commun. 2015 Oct 27;6:8692. doi: 10.1038/ncomms9692 (PMC4711745; doi:10.1038/ncomms9692)
Supplement: Supplementary Information — Supplementary Figures 1-9, Supplementary Table 1 and Supplementary References [file ncomms9692-s1.pdf]

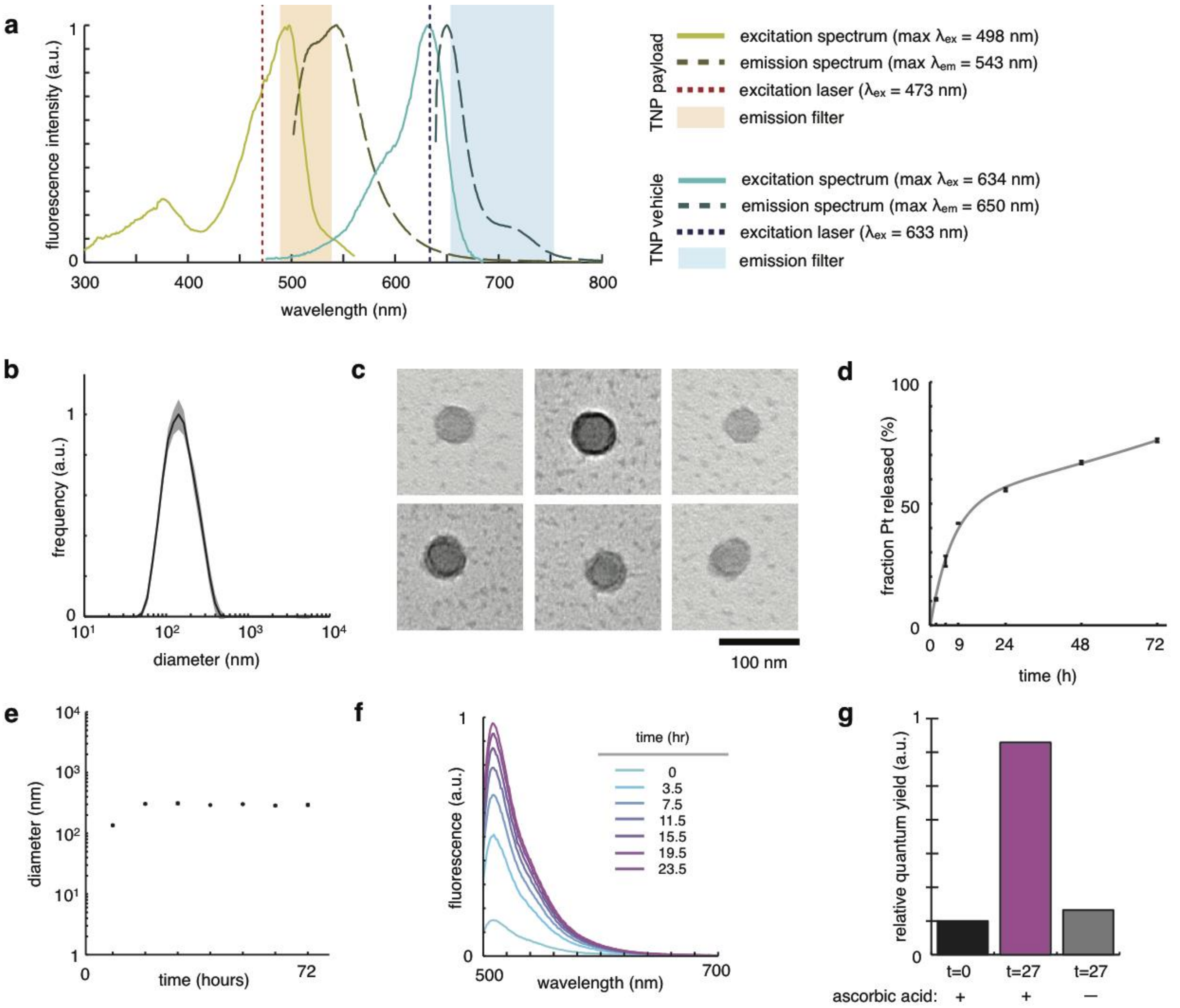

Supplementary Figure 1: TNP physicochemical characterization. (a) Excitation and emission spectra of the TNP vehicle and payload. (b) Dynamic light scattering (DLS) measurements of TNP diameter (line and shaded area denote mean ± S.E.M.; n=12). (c) Transmission electron microscopy (TEM) of TNP. (d) Kinetics of Pt-payload release from TNP into PBS at 37°C, measured by AAS (data are means ± S.E.M.; n=2). (e) Average TNP diameter as a function of incubation time in PBS at 37°C, measured by DLS (data are means ± S.E.M.; n=2). (f) Fluorescence turn-on upon Pt-reduction by incubation with 300 µM ascorbic acid in PBS. (g) Quantification of data in ***f*,** showing 6.3-fold fluorescence turn-on after 24 hr ascorbic acid incubation.

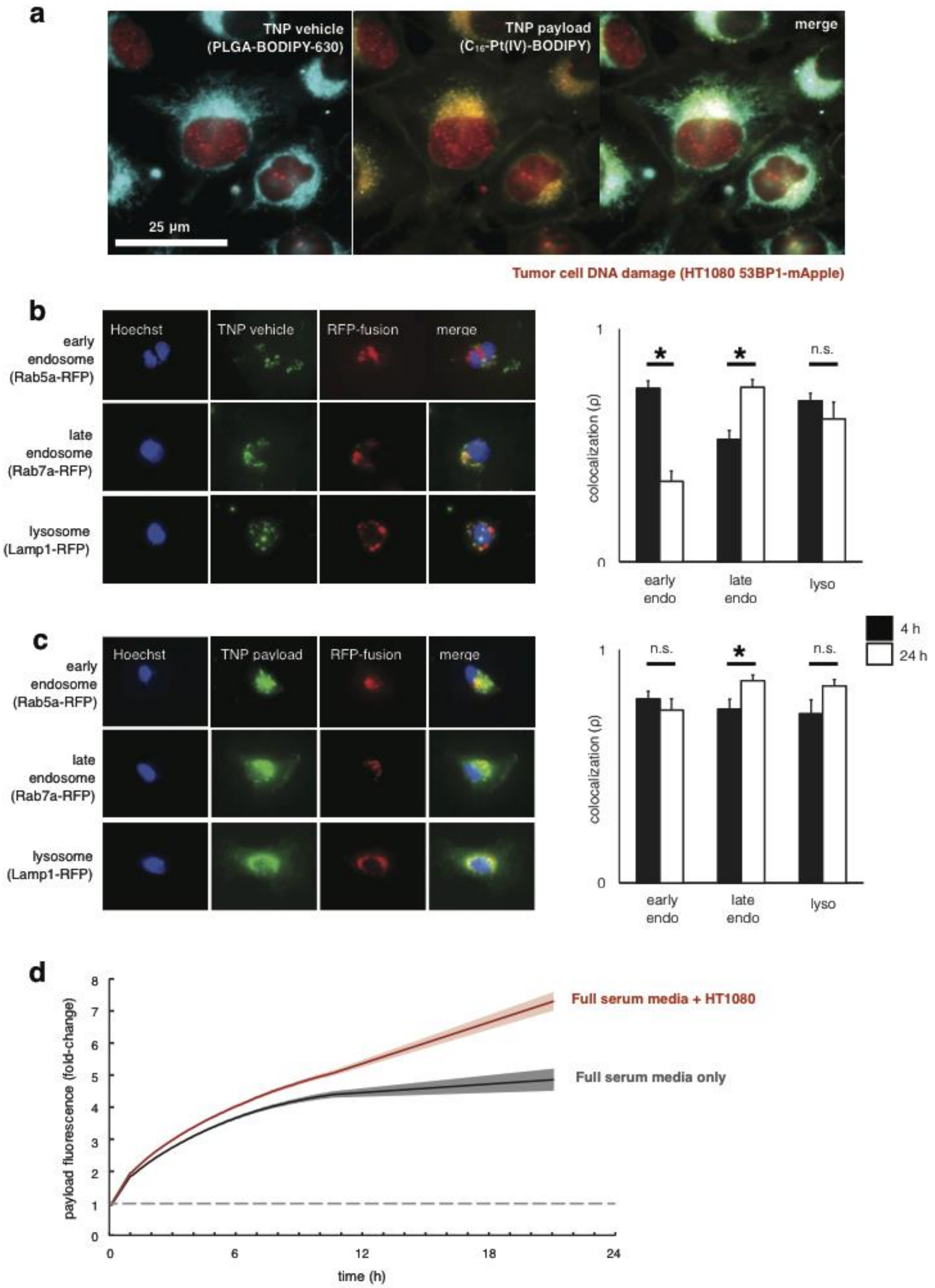

Supplementary Figure 2: Characterization of cellular TNP uptake. (a) Intracellular uptake of TNP after 24 hr incubation at 1 µM in live cells. (b-c) Co-localization analysis of TNP vehicle (b) and payload (c) with fluorescent proteins localized to endosomal and lysosomal compartments. Correlation was quantified over n=3 replicate plates, *$p<0.05$, two-sided t-test (means ± S.E.M.). (d) Payload fluorescence increases over time as it reduces from Pt(IV) to Pt(II). Lines and shaded area denote means ± S.E.M. (n=12).

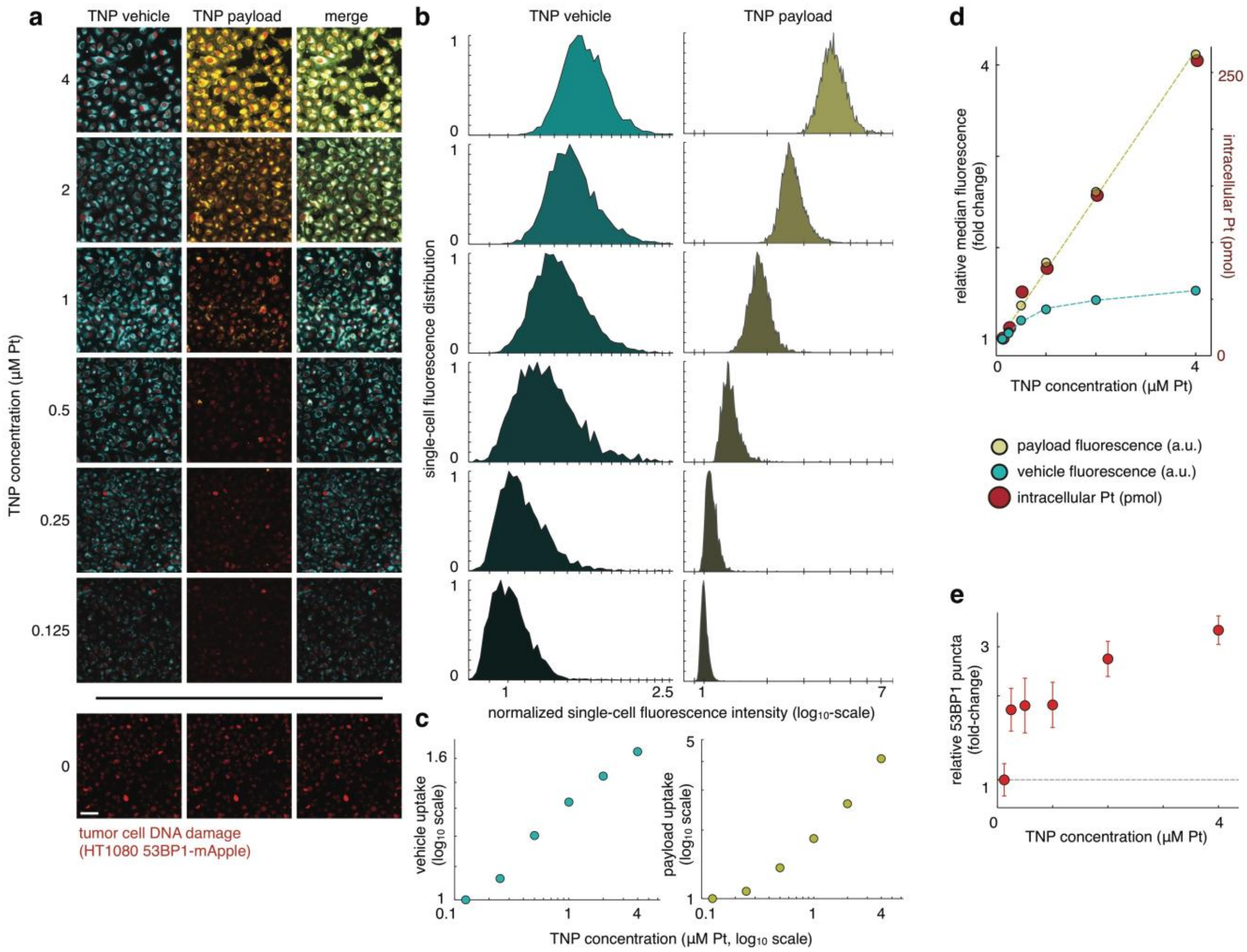

Supplementary Figure 3: TNP imaging calibration curves. (a) Images of TNP uptake were taken 24 hr following incubation. Scale bar = 50μm. (b) Single-cell TNP uptake was quantified after background-subtraction and normalization to the lowest concentration median (n > 16 replicate images). Plots correspond by row to sample images in ***a***. (c) Log-log plot of TNP fluorescence as a function of dose, corresponding to ***b***. Note the difference in slopes between vehicle and payload. (d) Linear plot of TNP fluorescence (left axis) as a function of dose (bottom axis) and intracellular Pt content, measured by AAS (right axis; n=3). The linear relationship between payload fluorescence and intracellular Pt content (R^2>0.99) supports their interconversion. (e) Relative DNA damage, measured by the fraction of cells exhibiting high (>5) 53BP1 puncta, and normalized to the untreated control, corresponding to dataset in ***a*** (means ± S.E.M.; n=3).

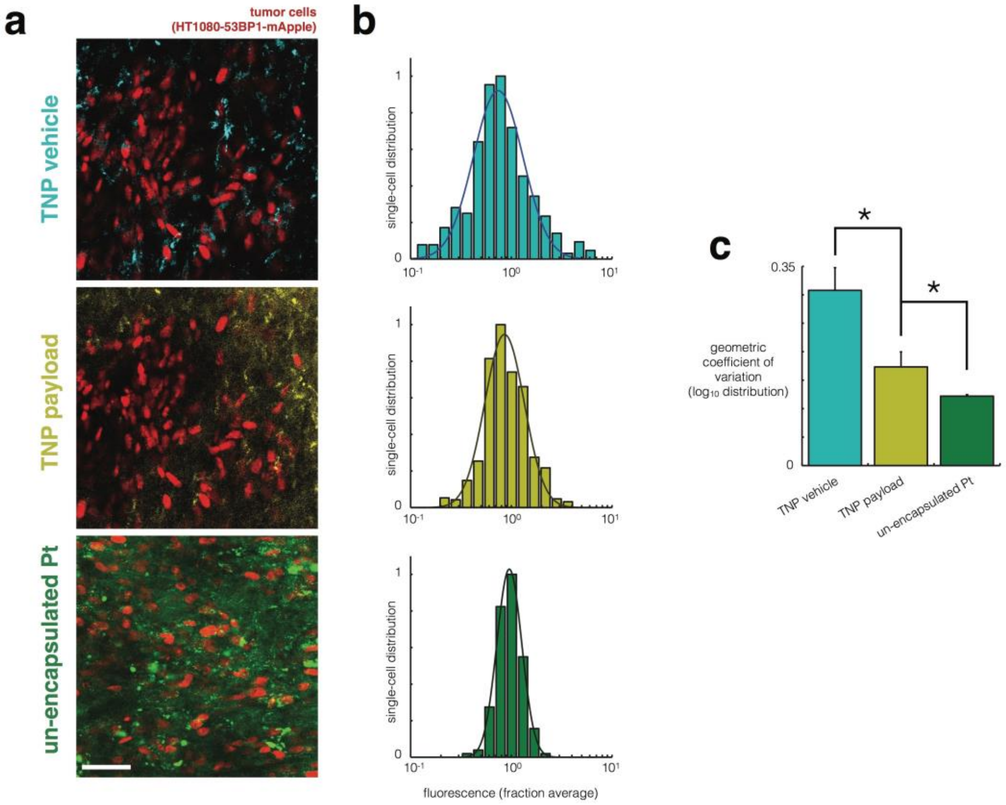

Supplementary Figure 4: TNP exhibits spatially heterogeneous accumulation within bulk tumor tissue. (a) Example images showing intratumoral distribution of TNP and unencapsulated BODIPY-labeled Pt(II), measured approximately 20 plasma half-lives post-injection. Scale bar = 50μm. (b) Distribution of fluorescence concentrations measured across single cells, mean-centered and log-transformed (n>200 cells / group across n=6 animals), corresponding to ***a***. Note the wider distribution for TNP vehicle. By comparison, the unencapsulated Pt(II) compound (CP-11) [1] exhibits more homogeneous accumulation. (c) Quantification of variance in ***b*** (*$p<0.05$, two-sided t-test; data are means ± S.E.M.; n≥2).

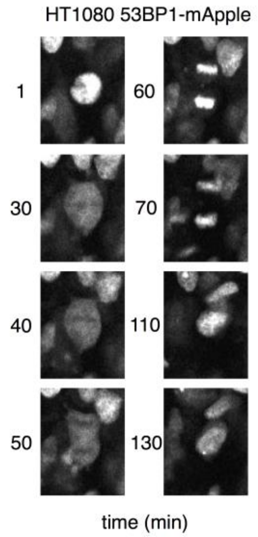

Supplementary Figure 5: Intravital imaging supports the real-time tracking of cell division events. In HT1080 xenografts, time-lapse confocal fluorescence microscopy allowed for the tracking of individual tumor cells over the course of 3 hr following injection of either TNP or the vehicle control. Cell division could then be identified in real time, shown here by example time-lapse images of an actively dividing cell monitored in the absence of TNP treatment.

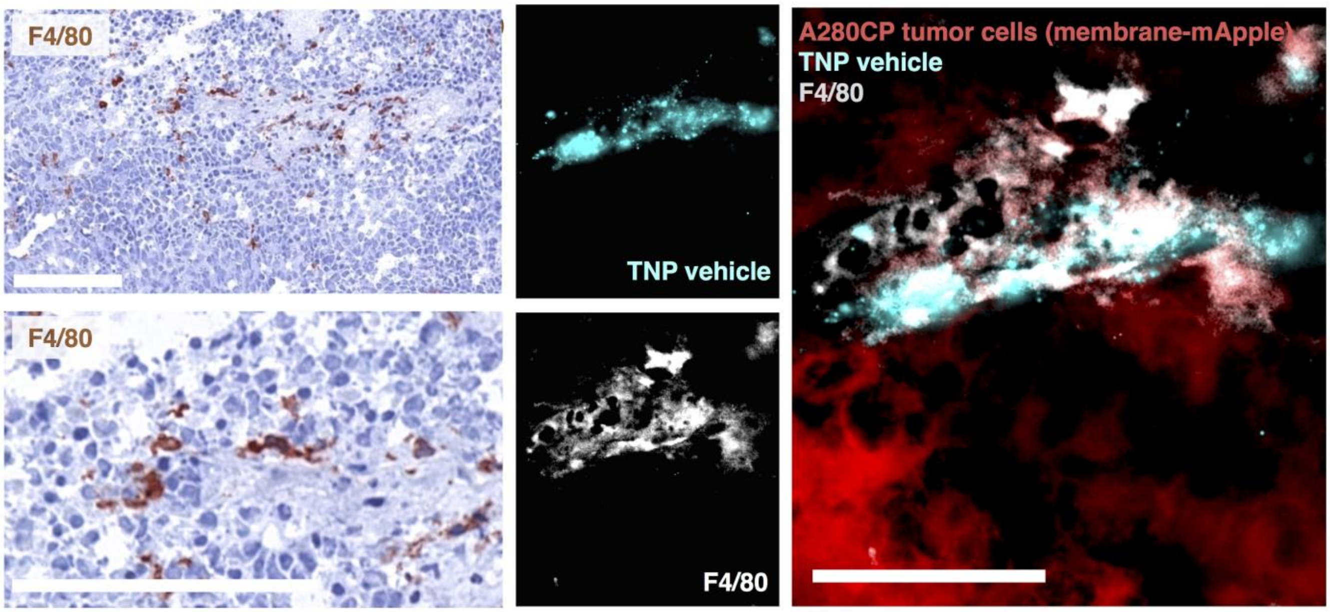

Supplementary Figure 6: TNP vehicle uptake in OVCA-associated F4/80+ host phagocytes. *Left:* 10x (top) and 40x (bottom) magnification images of immunohistochemistry, showing A2780CP tumors stained with hematoxylin and F4/80 (brown). Scale bar = 100µm. Immunohistochemistry corresponds with immunofluorescence (*middle/right)* of host phagocytes (stained for F4/80), tumor cells, and local accumulation of fluorescent TNP vehicle. Scale bar = 25 µm. OVCA cells (A2780CP) expressed membrane-tagged fluorescent protein (mApple). This representative histology provides further evidence that TNP vehicle is taken up by host phagocytes. F4/80 is one of several immunological markers used to identify TAMs as done here with flow cytometry (Fig. 5), which also shows significant TAM uptake of TNP vehicle.

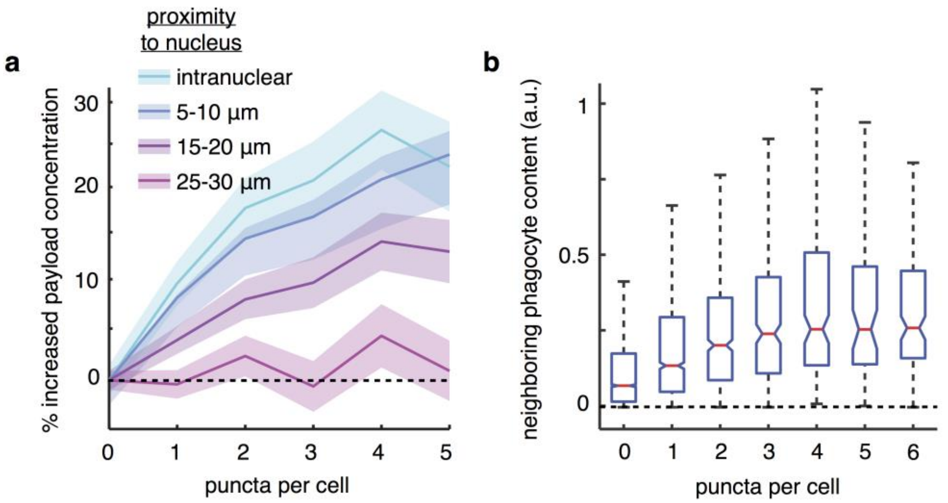

Supplementary Figure 7: Automated intravital imaging analysis shows that local phagocyte populations correlate with local TNP accumulation and tumor-cell DNA damage. (a) In HT1080 tumors, correlation between DNA damage and payload concentration significantly diminishes beyond 25 μm from cell nuclei (n>6000 cells; n=5 animals). Intravital images 3 hr after TNP injection were analyzed for single-cell DNA damage response (using 53BP1 puncta); automated image segmentation then computed correlation between DNA damage and corresponding local levels of payload fluorescence. Lines and shading denote means ± S.E.M. (b) Levels of single-cell DNA damage response correlate with the local accumulation of phagocytes that accumulate significant TNP-vehicle within 15 μm of each tumor cell nucleus (n>6000 cells; n=5 animals). Plots show boxes (25th and 75th quartiles), whiskers (10th and 90th percentiles), and medians (center line).

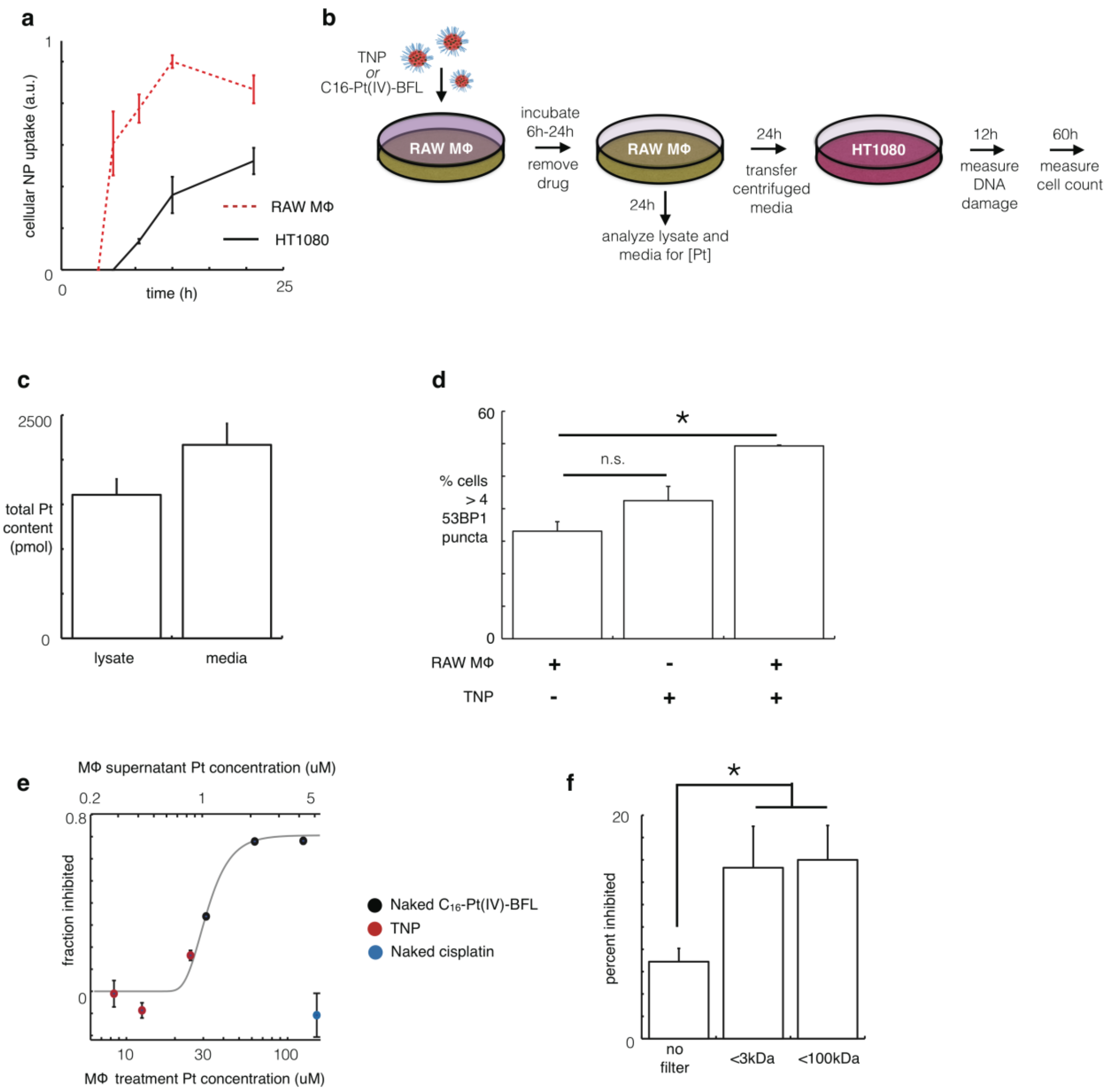

Supplementary Figure 8: TNP uptake in macrophages leads to increased Pt payload concentration in cell supernatant, which exhibits cytotoxic and DNA damaging capability. (a) Macrophages take up TNPs more rapidly and to a higher degree than tumor cells, as quantified by fluorescence and normalized to cell count (means ± S.E.M.; n=4). (b) Schematic of conditioned-medium experiments involving treatment of macrophages with TNPs, followed by analysis or transfer of conditioned medium to tumor cells. (c) Macrophages treated with TNP for 24 hr and then incubated in fresh medium for 24 hr ultimately release a substantial amount of Pt into the supernatant, as measured by AAS (means ± S.E.M.; n=3). (d-e) Transfer of Pt-containing conditioned medium from macrophages to tumor cells elicits an increase in DNA damage response after 24 hr (d) and reduces the number of live tumor cells after 72 hr (e) (means ± S.E.M.; n=3). (f) Size-exclusion filtration with either a 3 kDa or 100 kDa molecular weight cut-off filter does not reduce the cytotoxic impact of conditioned Pt-containing macrophage medium upon transfer to tumor cells, but rather increases it (p=0.005, two-tailed t-test, n = 5).

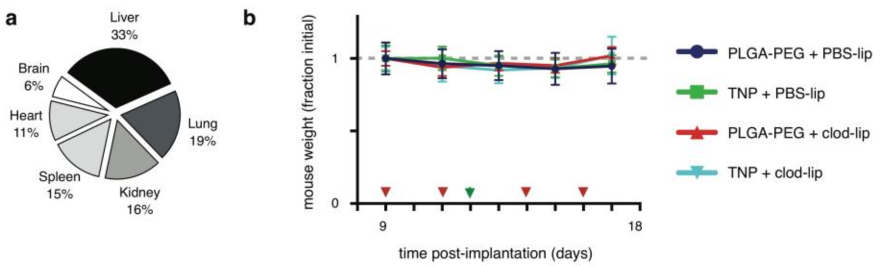

c

Cisplatin

Pt dose (mg/kg)	Blood Urea Nitrogen BUN (mg/dL)	Globulin GLOB (g/dL)	Aspartate Amino Transferase AST (U/L)	Cholesterol CH (mg/dL)	Creatinine CRT (mg/dL)
0	18	2	88.3	154.7	<0.2
1	21	2	83	138.3	<0.2
3	32	1.7	105	149	n.a.
5	20	1.8	51	133.7	<0.2

TNP

Pt dose (mg/kg)	Blood Urea Nitrogen BUN (mg/dL)	Globulin GLOB (g/dL)	Aspartate Amino Transferase AST (U/L)	Cholesterol CH (mg/dL)	Creatinine CRT (mg/dL)
0	18	2	88.3	154.7	<0.2
1	20.3	2.1	100	140.3	<0.2
3	21.5	2	140	136.7	<0.2
5	17.3	1.8	147	127.3	<0.2

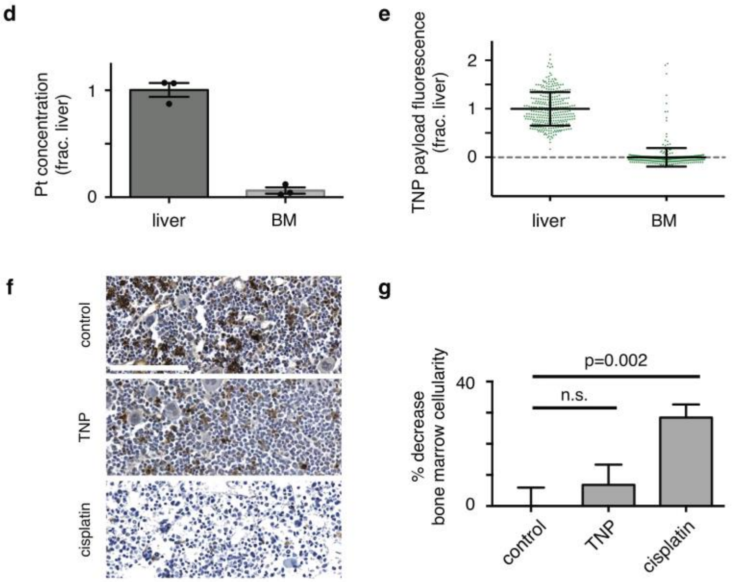

Supplementary Figure 9: TNP safely accumulates in the liver. (a) Pt accumulates most in the liver, 24 hr following i.v. TNP administration in male rats, measured by AAS (modified from Dhar et al.[2]). (b) TNP administration does not significantly impact mouse body weight, corresponding to treatment conditions in Fig. 8a (1 mg kg^{-1} Pt dose). Animals were treated with TNP (green arrow) and clodronate liposomes (red arrows) to systemically deplete macrophages (means ± S.E.M.; n≥9). (c) Therapeutic TNP doses do not significantly impact clinical chemistry measurements in Swiss albino mice (n=3). (d-e) TNP payload accumulation in the bone marrow (BM) is much less than with liver, measured by AAS (d) and C_{16}-Pt^{IV}-BODIPY fluorescence (e) (means ± S.E.M.; n=3). (f-g) Clinically equivalent i.v. dose of non-encapsulated cisplatin (7 mg kg^{-1}; [3]) causes significant loss of nucleated BM cell density, but while TNP does not. >10 fields of view (f) were quantified across n≥2 animals per group using automated CellProfiler analysis, quantified in *g* (*two-tailed t-test). TUNEL staining (brown) was also performed, but no statistically significant differences were observed across the 3 groups (n≥2). Scale bar = 100 µm.

	Un-encapsulated Pt	Nano-encapsulated Pt
**HT1080 IC_{50} (μM)**	1.7 ± 0.1	1.7 ± 0.02 (TNP)<br>3.7 ± 0.1 (un-encapsulated payload)
**OVCA429 IC_{50} (μM)**	15 ± 1	1.3 ± 0.1
**SKOV3 IC_{50} (μM)**	3 ± 0.3	1.5 ± 0.3
**MDAMB231 IC_{50} (μM)**	21.5 ± 1	5.2 ± 0.2
**PACA02 IC_{50} (μM)**	5.3 ± 0.3	1.2 ± 0.01
**PANC1 IC_{50} (μM)**	5.2 ± 0.5	8.7 ± 1.9
**PANC02 IC_{50} (μM)**	9.1 ± 0.8	8.1 ± 1
**Circulation half-life (min)**	10 ± 5 (depending on Pt derivative)<br>[Miller et al., 2014][1]	55 ± 5 (payload)<br>61 ± 6 (vehicle)
**Tissue heterogeneity (CV of cellular uptake)**	12%	17% (payload)<br>31% (vehicle)
**Relative tumor uptake**	63% [Xu et al., 2013][4]	100% [Xu et al., 2013][4]
**Size (nm)**	n/a	135 ± 0.9
**PDI**	n/a	0.18 ± 0.01
**Zeta potential (mV)**	n/a	-23 ± 1.4
**Loading efficiency**	n/a	9.1%
**Encapsulation efficiency**	n/a	73%
**Ex/Em wavelengths (nm)**	n/a	498/543 (payload)<br>634/650 (vehicle)

Supplementary Table 1: Characterization of Pt compounds. IC_{50} measured by cell count 72 hr after drug treatment. IC_{50} of unencapsulated Pt (left column) corresponds to cisplatin. Initial circulation half-life and tissue heterogeneity were measured by time-lapse fluorescence imaging in live animals. Reported tumor uptake measured by Pt content within bulk tumor using AAS. Size, poly-dispersity index (PDI), and zeta potential all measured by dynamic light scattering (DLS). For all values, n≥2 ± standard error.

Supplementary References:

1. Miller, M. A., Askevold, B., Yang, K. S., Kohler, R. H. & Weissleder, R. Platinum compounds for high-resolution in vivo cancer imaging. *ChemMedChem* **9**, 1131-1135 (2014).
2. Dhar, S., Kolishetti, N., Lippard, S. J. & Farokhzad, O. C. Targeted delivery of a cisplatin prodrug for safer and more effective prostate cancer therapy in vivo. *Proc Natl Acad Sci U S A* **108**, 1850-1855 (2011).
3. Nakagawa, T. et al. Expression of copper-transporting P-type adenosine triphosphatase (ATP7B) correlates with cisplatin resistance in human non-small cell lung cancer xenografts. *Oncol Rep* **20**, 265-270 (2008).
4. Xu, X. et al. Enhancing tumor cell response to chemotherapy through nanoparticle-mediated codelivery of siRNA and cisplatin prodrug. *Proc Natl Acad Sci U S A* **110**, 18638-18643 (2013).
